# Potent Liver‐Tropic mRNA Lipid Nanoparticles: ApoE‐Mediated Delivery Through a Low‐Density Lipoprotein Receptor Independent Uptake Mechanism

**DOI:** 10.1002/adma.202517893

**Published:** 2025-11-29

**Authors:** Ashish Sarode, Christian Ortiz, Tadeh Derstepanian, Natalia Vargas‐Montoya, Priyal Patel, Nikita Khadse, Saikat Manna, Ryan Landis, Joseph Skaleski, Lianne Boeglin, Hongfeng Deng, Anusha Dias, Hong Wang, Debora Barreiros Petropolis, Barak Yahalom, Shrirang Karve, Frank DeRosa

**Affiliations:** ^1^ mRNA Center of Excellence Sanofi Waltham MA 02451 USA; ^2^ Genomic Medicine Unit Sanofi Waltham MA 02451 USA; ^3^ Alpha Preclinical 200 Westboro Road, North Grafton Worcester MA 01536 USA

**Keywords:** ApoE, gene therapy, helper lipid, LDLR, lipid nanoparticle, liver tropism, mRNA

## Abstract

The development of nucleic acid therapeutics using non‐viral delivery systems requires efficient payload delivery to target organs for higher potency and tolerability. While lipid nanoparticle (LNP) formulations influence biodistribution, cellular uptake, and therapeutic efficacy, underlying mechanisms remain incompletely understood. This study develops potent mRNA‐LNP formulations and investigates determinants of liver tropism using ornithine transcarbamylase (OTC) deficiency as a protein replacement therapy model. Systematic screening of ionizable and helper lipids, optimization of composition and process, and biophysical characterization identify a liver‐tropic helper lipid–1,2‐dierucoyl‐sn‐glycero‐3‐phosphoethanolamine (DEPE) that modulates LNP structure and apolipoprotein E (ApoE) binding, enhancing liver‐specific delivery. Analysis of ionizable lipid chemistry reveals its role in cellular uptake mechanisms, leading to the identification of a novel ionizable lipid designed with N‐(2‐Hydroxyethyl)piperazine‐N′‐(4‐butanesulfonic acid) (HEPBS) core that enables efficient delivery independent of the low‐density lipoprotein receptor (LDLR) pathway. The optimized formulation achieves robust dose responsiveness, sustained therapeutic expression, and favorable tolerability in preclinical models. Therapeutic levels of OTC protein expression are observed with minimal toxicity, as indicated by stable liver function markers and cytokine levels. These findings provide mechanistic insights and establish a platform for mRNA‐based protein replacement therapies, supporting broader applications in rare genetic diseases requiring hepatic gene expression.

## Graphical abstract

Helper and ionizable lipids play a crucial role in determining ApoE binding and subsequent liver tropism and LDLR‐mediated uptake. Ionizable lipids primarily govern the LDLR‐independent uptake pathway. This complementary interplay between lipid components ultimately governs LNP delivery performance and therapeutic efficacy in the liver.

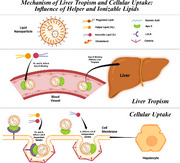

## 1 Introduction

Lipid nanoparticles (LNPs) have emerged as a transformative platform for nucleic acid delivery, with clinical validation exemplified by the success of mRNA‐based vaccines such as Comirnaty (BNT162b2), Spikevax (mRNA‐1273), mNEXSPIKE (mRNA‐1283), and mRESVIA (mRNA‑1345) in preventing infectious diseases.^[^ ^1^, ^2^, ^3^, ^4^, ^5^ ^]^ Similarly, Onpattro (patisiran)—the first FDA‐approved LNP‐formulated small interfering RNA (siRNA) therapeutic for hereditary transthyretin‐mediated amyloidosis (hATTR)—has established the therapeutic feasibility of LNP‐mediated nucleic acid delivery, although it is not classified as gene therapy.^[^ ^6^ ^]^ Thus, only five LNP‐based products have been FDA‐approved for clinical use in nucleic acid delivery to date—four as vaccines and just one as a therapeutic.^[^ ^7^ ^]^ In contrast, several recently approved gene therapies—including Zolgensma (for spinal muscular atrophy), Luxturna (for inherited retinal dystrophy), Kymriah (for hematologic malignancies), Skysona (for cerebral adrenoleukodystrophy), and Roctavian (for hemophilia A)—utilize viral vectors for in vivo gene transfer.^[^ ^8^ ^]^ Additionally, Casgevy, approved in late 2023 as the first CRISPR‐based gene‐editing therapy for sickle cell disease and transfusion‐dependent β‐thalassemia, employs ex vivo electroporation to deliver guide RNA and Cas9 protein into autologous hematopoietic stem cells.^[^ ^9^ ^]^ These examples highlight the substantial challenges associated with developing LNP‐based systems for therapeutic nucleic acid delivery, including achieving targeted biodistribution, ensuring cellular uptake by the appropriate cell populations, and optimizing key pharmacological attributes such as dose‐responsiveness, durability, repeat‐dosing potential, and overall tolerability.^[^ ^10^, ^11^, ^12^, ^13^ ^]^ Building upon the established clinical landscape in LNP‐based nucleic acid delivery and viral vector gene therapies, this work addresses key challenges limiting the broader application of LNPs as non‐viral delivery systems, particularly for treating genetic disorders affecting the metabolic functions of the liver.

The liver is a particularly attractive target for nucleic acid therapeutics—including mRNA, siRNA, antisense oligonucleotides (ASOs), closed‐ended DNA (ceDNA), and CRISPR‐Cas systems—due to its anatomical accessibility and central role in metabolic and systemic homeostasis.^[^ ^14^, ^15^, ^16^ ^]^ Hepatocytes express a broad repertoire of genes and are implicated in numerous monogenic disorders such as familial hypercholesterolemia, hemophilia, alpha‐1 antitrypsin deficiency, ornithine transcarbamylase (OTC) deficiency, and transthyretin amyloidosis.^[^ ^17^, ^18^, ^19^ ^]^ These characteristics make the liver an ideal target for gene silencing, protein replacement, and genome‐editing approaches. While hepatic delivery of ASOs and siRNA has been successfully achieved in clinical settings through conjugation strategies, more structurally complex nucleic acid therapeutics—including mRNA, ceDNA, and guide RNA required for protein replacement therapy, gene therapy, and CRISPR‐based genome editing—continue to rely on LNP formulation for effective liver delivery.^[^ ^20^ ^]^ Following intravenous administration, LNPs preferentially accumulate in the liver, facilitated by apolipoprotein E (ApoE) binding and subsequent uptake via low‐density lipoprotein receptor (LDLR)‐mediated endocytosis.^[^ ^21^, ^22^, ^23^, ^24^ ^]^ The surface properties and architecture of LNPs influence ApoE adsorption and thereby modulate liver tropism.^[^ ^24^ ^]^ This mechanistic understanding has driven the development of novel ionizable lipids, with studies demonstrating that lipid structure significantly impacts delivery efficiency.^[^ ^25^, ^26^ ^]^ Furthermore, formulation optimization through incorporation of cholesterol analogs,^[^ ^27^ ^]^ charged helper lipids,^[^ ^28^, ^29^ ^]^ and modified PEG‐lipids^[^ ^30^, ^31^ ^]^ has been explored to fine‐tune pharmacokinetic properties and biodistribution. Despite these advances, the structural and biophysical determinants of LNP behavior in vivo remain incompletely understood.^[^ ^32^ ^]^ We hypothesized that gaining mechanistic insights into how formulation components influence LNP architecture and biological performance would enable the rational design of delivery systems with improved hepatic specificity. Moreover, formulating LNPs that do not rely solely on ApoE or LDLR‐mediated uptake may be essential for treating genetic diseases such as familial hypercholesterolemia (FH), which often involves partial or complete LDLR deficiency.^[^ ^33^ ^]^

In this research work, we designed and synthesized novel ionizable lipids from two distinct chemical classes and systematically optimized their corresponding LNP formulations to elucidate key determinants of biodistribution and hepatic uptake. Critical roles for helper lipid selection and overall composition in modulating LNP structure and liver tropism were identified through comprehensive formulation screening, structural characterization, and in vivo protein expression studies. Notably, cellular uptake pathways were primarily influenced by ionizable lipid chemistry. A foundation for the rational development of next‐generation, liver‐directed delivery platforms was formed by these structure‐function insights. Leveraging this mechanistic insight, an optimized mRNA‐LNP formulation targeting OTC deficiency—a representative model for mRNA‐based protein replacement therapy—was developed. Building on single‐dose efficacy and tolerability demonstrated in mice, the formulation was evaluated for repeat dosing in Sprague–Dawley (SD) rats, a species selected for its higher sensitivity to LNP‐associated toxicity and established use in preclinical safety studies.^[^ ^34^ ^]^ This approach allowed assessment of therapeutic potency and safety parameters following multiple administrations—a critical aspect for transient protein replacement therapy.

OTC deficiency, an inherited urea cycle disorder caused by mutations in the OTC gene, leads to impaired ammonia detoxification and typically presents with hyperammonemia in infancy.^[^ ^35^ ^]^ The deficiency of OTC, a key mitochondrial enzyme catalyzing the reaction between ornithine and carbamoyl phosphate to form citrulline, results in neurotoxic ammonia accumulation, contributing to developmental delay, cognitive impairment, neurological dysfunction, and, in severe cases, fatality.^[^ ^36^ ^]^ Current treatment options—including dietary protein restriction, ammonia‐scavenging agents, dialysis, and supplementation with arginine or citrulline—are palliative and often insufficient to prevent metabolic crises. Liver transplantation remains the only curative approach but is limited by donor availability and associated risks, particularly in neonates and infants.^[^ ^37^, ^38^, ^39^ ^]^ As such, mRNA‐based protein replacement therapy represents a promising therapeutic alternative.^[^ ^40^ ^]^ Here, we report a novel LNP formulation encoding human OTC mRNA that demonstrates robust and transient protein expression in preclinical models, along with favorable pharmacological properties including dose responsiveness, desired duration of expression, repeat‐dosing compatibility, and favorable tolerability. Overall, our work has established a foundation for mRNA‐based protein replacement therapy through key mechanistic insights and innovative formulation approaches, offering new treatment possibilities for rare genetic diseases dependent on hepatic gene expression.

## 2 Results and Discussion

### 2.1 Structural Design and Screening of Diketopiperazine (DKP)‐Based Ionizable Lipids

**Figure 1:**
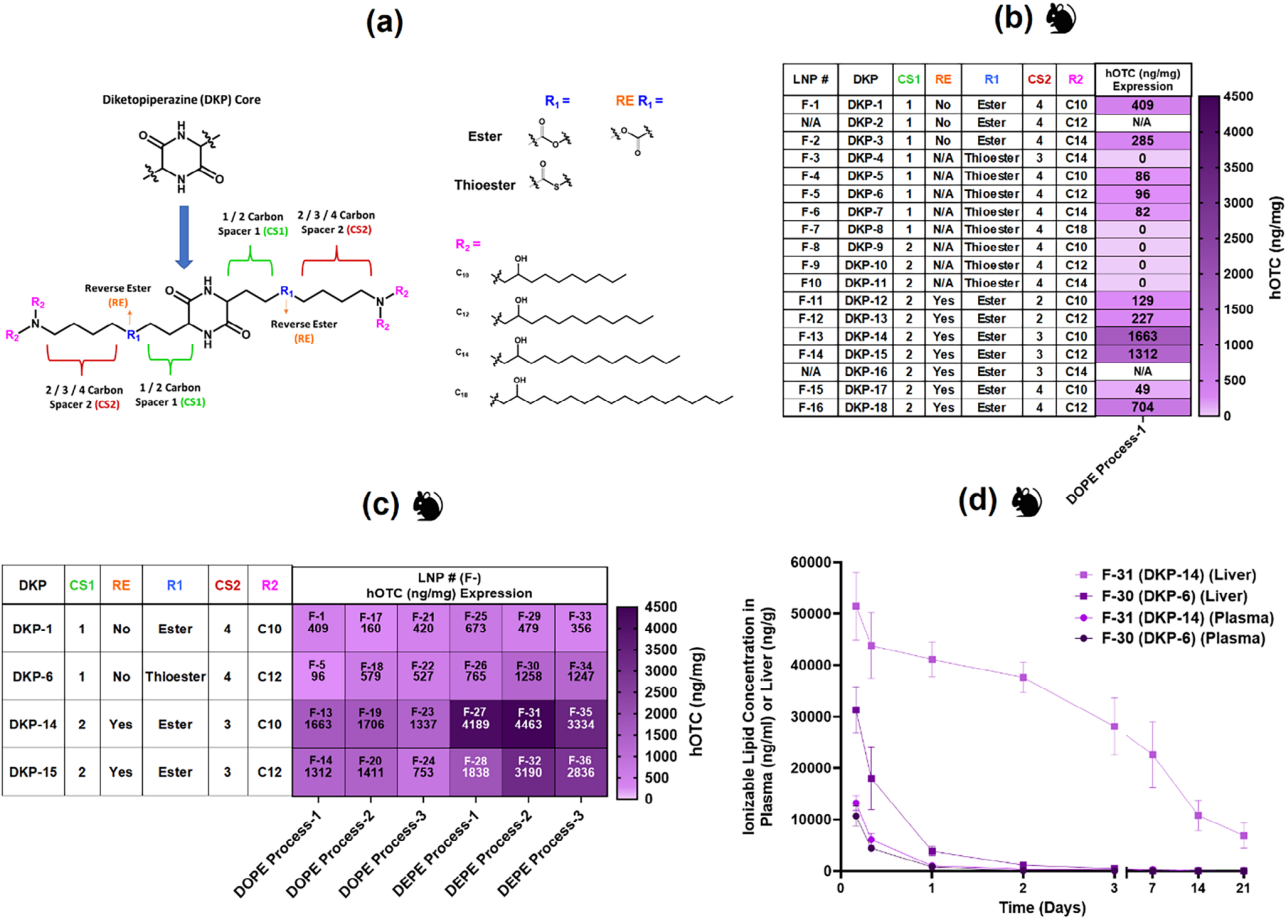
Structural Design and Screening of Diketopiperazine (DKP)‐Based Ionizable Lipids in Mice. a) Design of lipids featuring varying carbon spacer lengths and tertiary amine‐containing carbon tails with ester or thioester linkers adjoining the diketopiperazine core. b) Heat map depicting hOTC expression at 1 mg kg^−1^ mRNA dose in mice 24 h post‐administration of LNPs formulated with DKP lipids. Lipids with ester linkers demonstrated higher potency compared to their thioester analogs, with reverse carbonyl ester‐containing lipids exhibiting maximum potency. Carbon spacer chain length variations significantly impacted potency. c) Formulation optimization using lead DKP lipids. Protein expression was significantly enhanced with DEPE helper lipid compared to DOPE for all lead lipids across three processes. Process optimization improved the expression for select ionizable lipids. d) Favorable pharmacokinetic profile of ionizable lipid DKP‐6, indicating promise for repeat dosing applications, leading to the selection of LNP F‐30 as the lead formulation for further optimization.

The alkenyl amino alcohol ionizable lipid family containing DKP core was expanded to optimize human ornithine transcarbamylase (hOTC) expression, building upon previous work in which the efficacy of these lipids for mRNA delivery was demonstrated (**Figure** **1**a).^[^ ^41^, ^42^ ^]^ Variable‐length carbon spacers (CS1 and CS2), connected via ester or thioester linkages (R1), were systematically attached to both sides of the diketopiperazine core. Tertiary amine groups were positioned at CS2 termini, each functionalized with two lipid chains (R2) of varying lengths. Additionally, reverse ester (RE) analogs were synthesized for lipids containing ester‐based biodegradable linkers. LNP formulations were prepared using Process‐1 (Figure S1, Supporting Information), incorporating these ionizable lipids using composition‐4 and 1,2‐dioleoyl‐sn‐glycero‐3‐phosphoethanolamine (DOPE) as helper lipid, to establish structure‐activity relationships in mice.

Formulation constraints emerged with R2 chain lengths, such as lower encapsulation efficiency or colloidal instability, as successful LNP formation was limited to only 10‐14 carbon chains. Liver hOTC expression analysis (Figure 1b) revealed superior potency of ester‐linked lipids compared to their thioester counterparts, exemplified by higher expression from LNP formulation (F)–F‐1 and F‐2 vs F‐4 and F‐6. This differential performance likely stems from the enhanced biodegradability of thioester groups, resulting in reduced stability and consequently lower protein expression. Notably, reverse ester‐containing lipids (DKP‐14 and DKP‐15) demonstrated the highest potency, with LNPs F‐13 and F‐14 significantly outperforming all other formulations. Carbon spacer length critically influenced potency: thioester lipids showed optimal performance with one‐carbon CS1 spacers (F‐4, F‐5, F‐6) vs two‐carbon analogs (F‐8, F‐9, F‐10), while reverse ester lipids performed best with three‐carbon CS2 linkers (F‐13 and F‐14) compared to two‐ (F‐11 and F‐12) or four‐ (F‐15 and F16) analogs.

Lead candidates underwent further optimization using composition‐4, three manufacturing processes (1‐3), and two helper lipids–DOPE and 1,2‐dierucoyl‐sn‐glycero‐3‐phosphoethanolamine (DEPE) (Figure 1c). Process optimization enhanced expression for specific ionizable lipids, with DKP‐6 showing systematic improvements across processes 1‐3 with both helper lipids. Helper lipid selection proved more influential than the manufacturing process, with DEPE consistently outperforming DOPE across all formulations and processes, as we previously demonstrated.^[^ ^43^ ^]^ Pharmacokinetic evaluation of lead candidates F‐30 (DKP‐6) and F‐31 (DKP‐14) revealed rapid plasma clearance for both, but dramatically different liver clearance profiles (Figure 1d). While DKP‐6 cleared from the liver within ≈2 days, DKP‐14 persisted beyond 21 days—likely due to differential biodegradation rates of thioester vs ester linkages. Considering the requirements for repeat dosing applications, the rapidly clearing DKP‐6‐based LNP F‐30 was selected for further development to prevent potential hepatic accumulation.

### 2.2 Lead DKP Formulation: Establishment of Dose–Activity and Duration in Mice; Limited Dose–Ranging Outcomes in Rats

**Figure 2:**
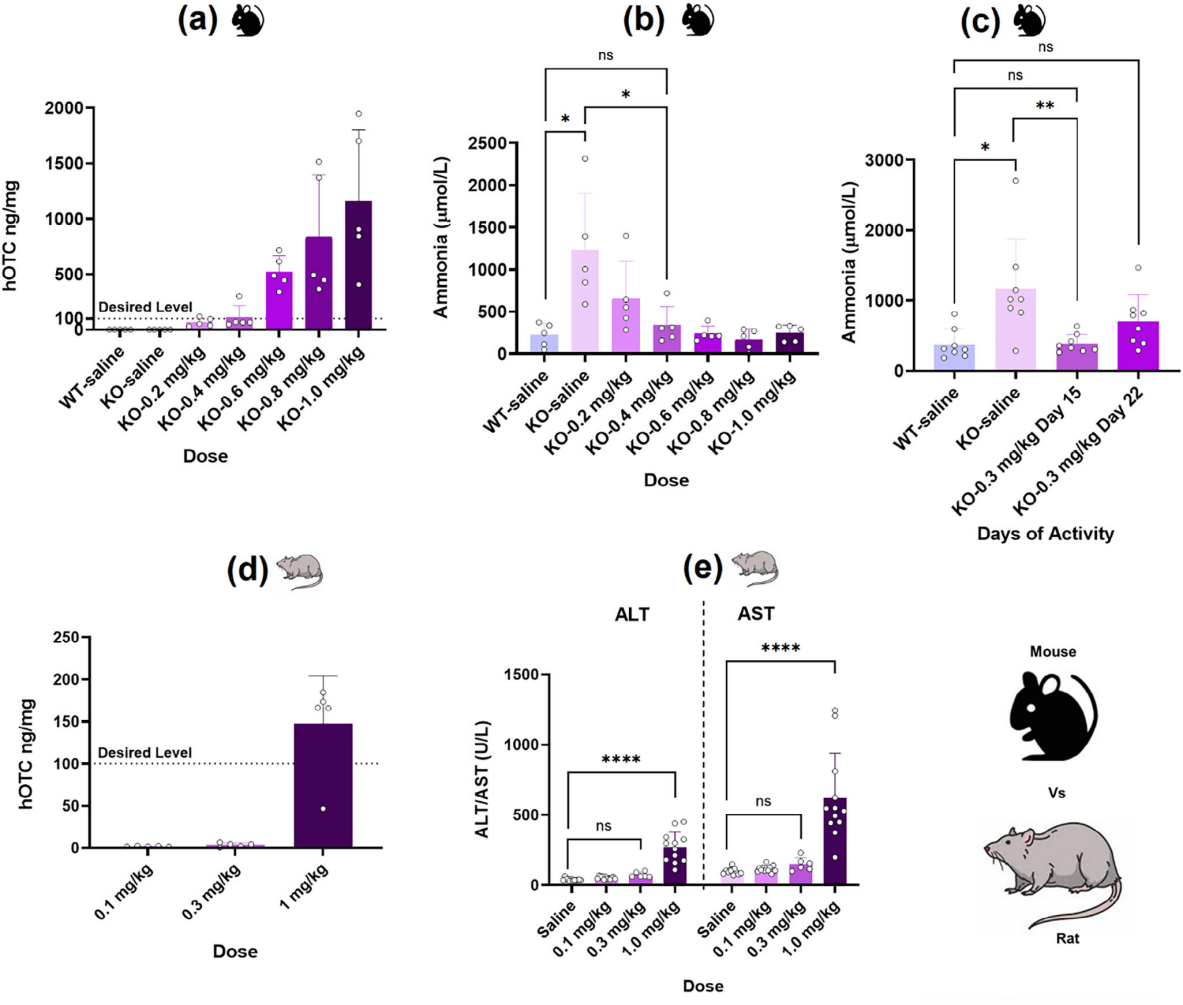
Lead DKP Formulation: Establishment of Dose–Activity and Duration in Mice; Limited Dose–Ranging Outcomes in Rats. The lead LNP formulation F‐30, containing ionizable lipid DKP‐6, demonstrated strong dose‐dependent activity and a desired duration of action. a) Dose‐dependent increase in hOTC expression in OTC knock‐out (KO) mice. b) Inverse relationship between ammonia levels and dose, corresponding to increased hOTC expression. hOTC expression of ≈100 ng mg^−1^, achieved between 0.2 and 0.4 mg kg^−1^ dose in KO mice, was identified as the desired level for sufficient ammonia metabolism comparable to wild‐type (WT) mice. c) Sustained ammonia metabolism for about three weeks at a 0.3 mg kg^−1^ dose. d) hOTC expression in rats exceeding the desired level of 100 ng mg^−1^ at a 1 mg kg^−1^ dose. e) Significant elevation of liver enzymes ALT and AST at 1 mg kg^−1^ dose in rats, indicating potential tolerability issues for chronic dosing regimens. Student's *t*‐Test with Welch's Correction, where *n* = 5 for (a), (b), and (d), *n* = 8 for (d), and *n* = 12 for (e). Asterisks denote *p*‐values: ^****^ *p* < 0.0001, ^***^ *p* < 0.001, ^**^ *p* < 0.01, ^*^ *p* < 0.05. ns denotes statistical insignificance.

Previously, Prieve et al. demonstrated normalization of ammonia levels in an OTC‐deficient mouse model–OTC^spf‐ash^ using a hybrid mRNA‑LNP/Polymer Micelle product (ARCT‑810 / LUNAR‑OTC), which translated to human clinical trials and is currently being evaluated in an ongoing Phase II study.^[^ ^44^, ^45^, ^46^ ^]^ Lead LNP formulation F‐30 from the DKP family was evaluated in the same hOTC knockout (KO) mouse model‐OTC^spf‐ash^ (**Figure** **2**a). hOTC expression increased linearly with escalating dose levels, establishing a clear dose‐response relationship. Importantly, this protein expression correlated inversely with ammonia levels, which decreased proportionally with increasing hOTC expression (Figure 2b). This established a clear relationship between dose, protein expression, and activity for the formulation in mice. Ammonia levels in hOTC KO mice receiving doses ≥0.4 mg kg^−1^ were comparable to those in wild‐type (WT) mice, indicating normalized ammonia metabolism. While the 0.2 mg kg^−1^ dose reduced ammonia levels compared to saline‐treated KO mice, these remained elevated relative to WT animals. Based on these findings, a threshold hOTC expression level of 100 ng mg^−1^ was identified as necessary for sufficient ammonia metabolism, corresponding to the 0.4 mg kg^−1^ dose (Figure 2a). However, to optimize the formulation for repeat dosing while maintaining tolerability, a slightly lower dose of 0.3 mg kg^−1^ was selected for duration of action studies in hOTC KO mice. The 0.3 mg kg^−1^ dose of F‐30 LNP maintained therapeutic activity for approximately three weeks (Figure 2c). Ammonia levels in KO mice at 15‐ and 22‐days post‐administration were comparable to saline‐treated WT mice and significantly lower than saline‐treated KO mice. These results demonstrated that the 100 ng mg^−1^ hOTC expression achieved at 24 h post‐dosing effectively controlled ammonia levels for 2–3 weeks, suggesting the feasibility of biweekly or triweekly repeat dosing regimens.

Following a successful duration of action studies in mice, dose‐ranging studies were conducted in rats. Since no hOTC KO rat model exists, the goal was set on achieving target hOTC expression levels. F‐30 LNP administered at 0.1, 0.3, and 1 mg kg^−1^ (Figure 2d) showed that only the 1 mg kg^−1^ dose achieved the desired expression threshold of 100 ng mg^−1^. However, this dose exhibited poor tolerability, with significantly elevated liver biomarkers–alanine aminotransferase (ALT) and aspartate aminotransferase (AST) compared to saline controls, while the 0.3 mg kg^−1^ dose maintained normal biomarker levels (Figure 2e). These findings necessitated screening alternative ionizable lipid families to develop more potent formulations capable of achieving sufficient expression in rats at tolerable doses. Despite process optimization benefits observed with DKP‐6, other lipids in this family showed inconsistent expression patterns across manufacturing processes (Figure 1c). Consequently, conventional process‐4 was selected for subsequent screening. Additionally, to mitigate potential anti‐PEG antibody formation during repeat dosing, 1,2‐Dimyristoyl‐rac‐glycero‐3‐methoxypolyethylene glycol‐2000 (DMG‐PEG‐2000) content was reduced to 2 mol%. This modification did not significantly alter hOTC expression in DKP‐6 LNPs with either DOPE or DEPE helper lipids (Figure S2, Supporting Information).

### 2.3 Structural Design and Screening of GOOD Asymmetric (GA) Ionizable Lipids

**Figure 3:**
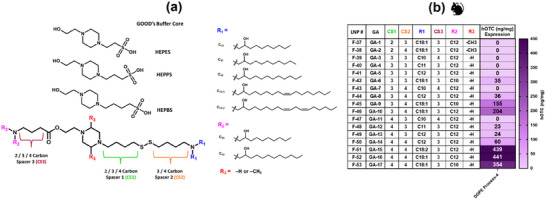
Structural Design and Screening of GOOD Asymmetric (GA) Ionizable Lipids in Mice. a) Design and synthesis of asymmetric ionizable lipids based on piperazine cores from Norman Good's buffers (HEPES, HEPPS, and HEPBS). The structures feature hydroxy ethyl and alkane sulfonic acid moieties modified with ester and disulfide linkages, respectively, combined with varying carbon spacer lengths and tertiary amine‐containing carbon tails adjoining the piperazine core. b) Heat map showing hOTC expression at 0.5 mg kg^−1^ mRNA dose in mice 24 h post‐administration of LNPs formulated with GA lipids. Significant potency enhancement was achieved using longer, unsaturated lipid tails, leading to the identification of GA‐15 and GA‐16 as lead ionizable lipids for formulation optimization.

We have designed a novel class of ionizable lipids based on piperazine cores of some of Norman Good's buffers, as described by Karve et. al.^[^ ^47^ ^]^ **Figure** **3**A shows the three distinct cores: 4‐(2‐hydroxyethyl)‐1‐piperazineethanesulfonic acid (HEPES), 3‐[4‐(2‐Hydroxyethyl)piperazin‐1‐yl]propane‐1‐sulfonic acid (HEPPS), and N‐(2‐Hydroxyethyl)piperazine‐N'‐(4‐butanesulfonic acid) (HEPBS) that were used for designing these lipids. The characteristic piperazine structure was maintained in each core, with hydroxyethyl and alkane sulfonic acid groups substituted at the 4‐ and 1‐nitrogen positions, respectively. This structure enabled the incorporation of degradable ester and disulfide moieties, along with carbon spacer and asymmetric lipid tails on both arms of the molecule. Specifically, a disulfide linkage connected two variable carbon spacers (CS1 and CS2), while a third spacer (CS3) was positioned at the terminus of the biodegradable ester linkage. The termini of CS2 and CS3 were functionalized with tertiary amine groups carrying variable‐length lipid chains R1 and R2, respectively. Building upon our previous successful application of HEPES core‐based lipids for intramuscular vaccine delivery,^[^ ^48^ ^]^ modifications to the piperazine core were introduced at the 2‐ and 5‐carbon positions with methyl substitutions for intravenous administration. LNPs of these ionizable lipids formulated using process‐4 and composition‐3 with DOPE as a helper lipid were screened for hOTC expression at a dose of 0.5 mg kg^−1^ (Figure 3b). Notably, methylated HEPES core‐based lipids showed no detectable protein expression, prompting further investigation of only HEPPS and HEPBS cores. Structure‐activity relationship analysis revealed that carbon spacer length significantly influenced protein expression. Systematic extension of CS1 and/or CS2 progressively enhanced hOTC expression, as demonstrated by LNPs F‐41, F‐44, F‐49, and F‐50 formulated with GA‐5, GA‐8, GA‐13, and GA‐14, respectively. While F‐41 (containing GA‐5 with 3‐carbon CS1 and CS2) showed no detectable expression, single‐carbon extensions in either CS2 (GA‐8) or CS1 (GA‐13) yielded measurable hOTC expression in F‐44 and F‐49. Further enhancement occurred when both spacers were extended simultaneously, as seen with GA‐14 in F‐50. This pattern was more pronounced with GA‐6, GA‐9, and GA‐17 formulations (F‐42, F‐45, and F‐53), where expression increased in the order GA‐17 >GA‐9 >GA‐6, correlating with incremental carbon chain extensions in CS1 and CS2. Tail length modifications at the ester‐side tertiary amine also influenced expression, with 12‐carbon R2 chains (GA‐16/F‐52 and GA‐10/F‐46) outperforming their 10‐carbon analogs (GA‐17/F‐53 and GA‐9/F‐45). Most significantly, incorporating longer unsaturated tails at the disulfide‐side tertiary amine substantially enhanced expression, as evidenced by GA‐16/F‐52 and GA‐10/F‐46 vs their structural analogs GA‐8/F‐44 and GA‐14/F‐50. Interestingly, increasing unsaturation from one double bond (GA‐16) to two (GA‐15) produced no significant improvement. Based on promising hOTC expression levels, GA‐15 and GA‐16 were identified as lead candidates for subsequent formulation optimization.

### 2.4 Optimization of Protein Expression

#### 2.4.1 Effects of Ionizable Lipids, Helper Lipids, and LNP Composition

**Figure 4:**
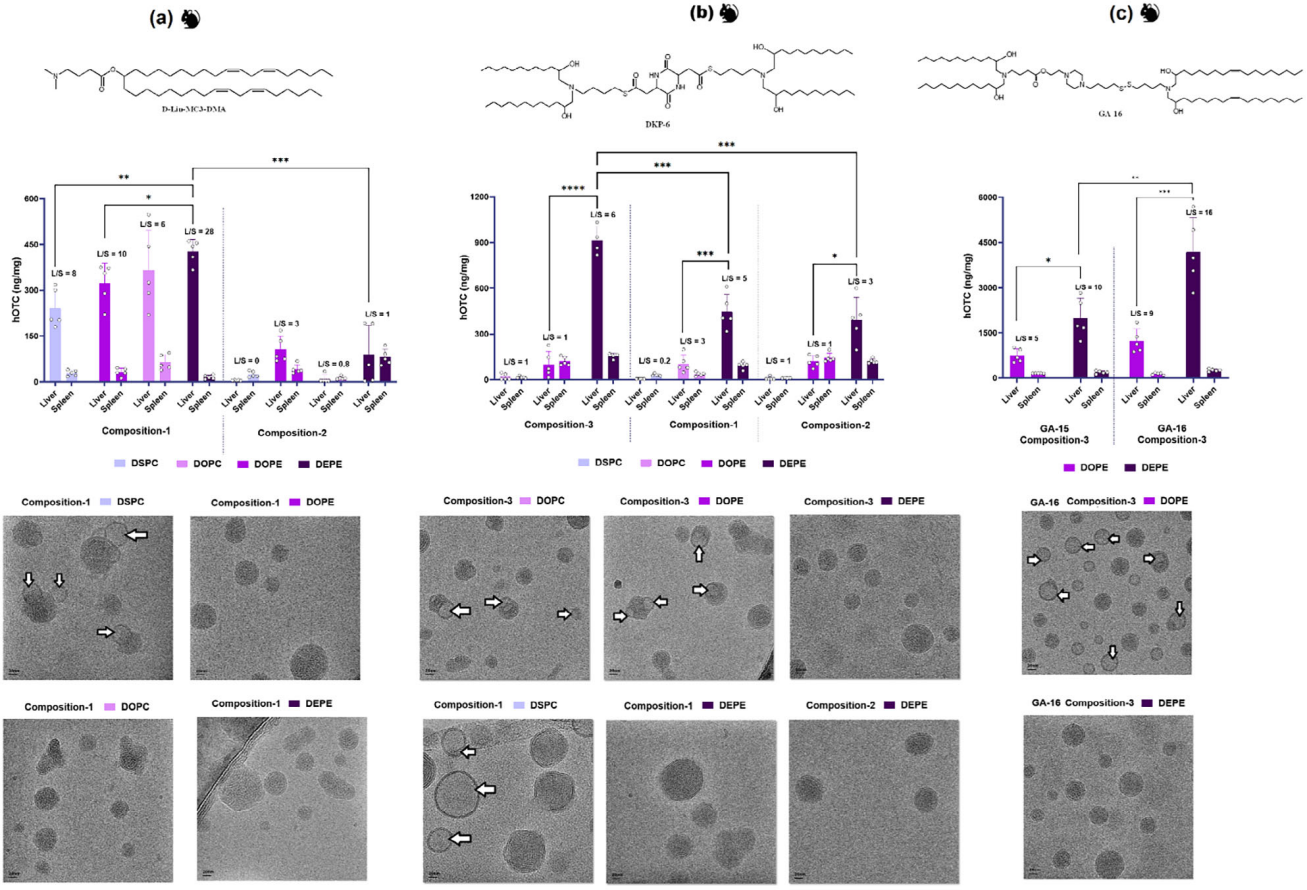
Optimization of Protein Expression in Mice: Effects of Ionizable Lipids, Helper Lipids, and LNP Composition on LNP Structure. hOTC expression plots at 1 mg kg^−1^ mRNA dose in mice and cryo‐TEM images of LNPs formulated with three ionizable lipids using various helper lipids and compositions. a) DLin‐MC‐3‐DMA (MC‐3) served as the benchmark control. b) DKP‐6, selected from the DKP family. c) GA‐15 and GA‐16, selected from the GA family. Across all ionizable lipids tested, DEPE‐based LNPs demonstrated the highest hOTC expression compared to other helper lipids. This enhanced performance correlated with the homogeneous, predominantly multi‐lamellar structures observed in the corresponding cryo‐TEM images of DEPE‐based LNPs. Composition‐1 yielded optimal expression for MC‐3, while composition‐3 proved most effective for DKP‐6. Student's *t*‐Test with Welch's Correction (*n* = 5). Asterisks denote *p*‐values: ^****^ *p* < 0.0001, ^***^ *p* < 0.001, ^**^ *p* < 0.01, ^*^ *p* < 0.05.

A comprehensive formulation optimization study was conducted to elucidate the influence of helper lipids and compositional variations on protein expression. Lead ionizable lipids DKP‐6, GA‐15, and GA‐16 were formulated using process‐4 with four helper lipids 1,2‐Distearoyl‐sn‐glycero‐3‐phosphocholine (DSPC), 1,2‐dioleoyl‐sn‐glycero‐3‐phosphocholine (DOPC), DOPE, and DEPE. Similarly, using the above helper lipids, LNP formulations with DLin‐MC‐3‐DMA (MC‐3) as a benchmark ionizable lipid were also evaluated. MC‐3 was selected since it represents the ionizable lipid component in the only FDA‐approved LNP product currently employed for therapeutic liver‐targeted delivery of nucleic acids in clinical applications. Three different LNP compositions with varying molar ratios of lipid components (**Table** **1**) were systematically evaluated. Among all formulations and ionizable lipids tested, DEPE‐containing LNPs consistently demonstrated higher hOTC expression levels (**Figure** **4**). For MC‐3‐based formulations (Figure 4a), composition‐1 outperformed composition‐2 regardless of helper lipid selection. Performance of helper lipids followed a clear trend: DSPC < DOPE ≤ DOPC < DEPE, with DEPE yielding maximal hOTC expression. Similarly, DKP‐6 formulations showed high expression with DEPE across all compositions, maintaining the same trend (DSPC < DOPC ≤ DOPE < DEPE) as shown in Figure 4b. Among the compositions tested with DKP‐6, composition‐3 demonstrated higher expression, while compositions‐1 and −2 yielded comparable results when formulated with DEPE. Based on the higher expression of composition‐3 with DEPE for the lipidoid‐like DKP‐6 structure, GA‐15 and GA‐16 were also evaluated with the same composition. As illustrated in Figure 4c, DEPE‐containing LNPs consistently outperformed their DOPE counterparts for both GA‐15 and GA‐16, with GA‐16 formulations achieving significantly higher hOTC expression than GA‐15 across both helper lipids. Notably, the lead GA‐16/DEPE formulation achieved a very high hOTC expression level of ≈4200 ng mg^−1^, substantially exceeding those of optimized DKP‐6 (900 ng mg^−1^) and MC‐3 (420 ng mg^−1^) formulations. In addition to the high expression levels, DEPE formulations also demonstrated superior liver‐to‐spleen (L/S) ratios across all ionizable lipids tested.

**Table 1:** Various molar compositions are used for ionizable lipids screening and formulation optimization.

Composition	Mole percent of lipid components			
	DMG‐PEG‐2000	Ionizable lipid	Cholesterol	Helper lipid
1	1.5	50	38.5	10
2	1.5	40	28.5	30
3	2	40	25	33
4	3	40	25	32

**Figure 5:**
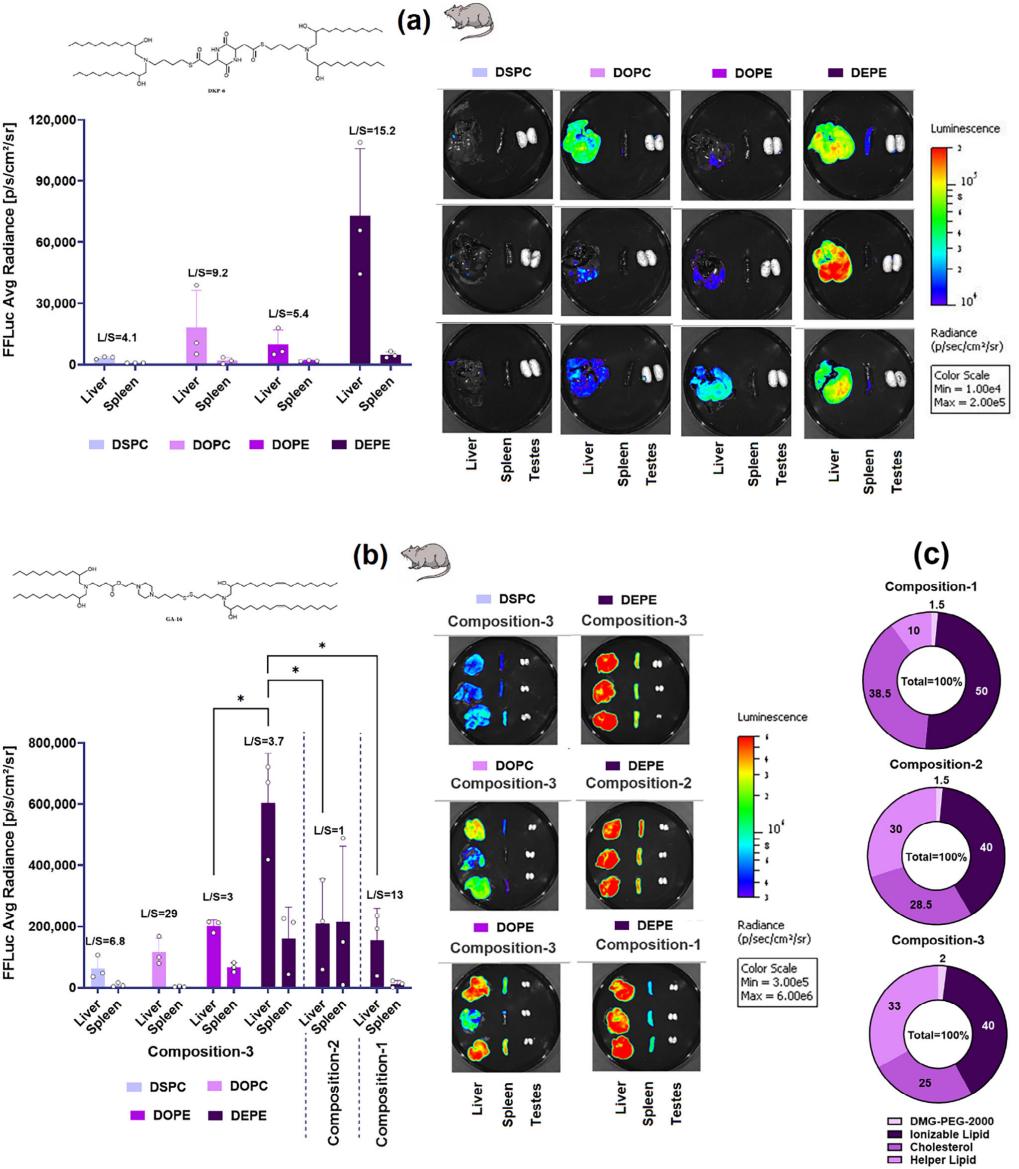
Optimization of Protein Expression in Rats: Effects of Ionizable Lipids, Helper Lipids, and LNP Composition. a) Evaluation of DKP‐6, selected from the DKP family. b) Evaluation of GA‐16, selected from the GA family. c) Percent molar ratios of various compositions explored for GA‐16. Consistent with mouse studies, DEPE‐based LNPs demonstrated superior FFLuc expression at 0.03 mg kg^−1^ mRNA dose in rats compared to formulations with alternative helper lipids. Composition‐3 yielded the highest FFLuc expression for GA‐16, further confirming the trends observed across species. Student's *t*‐Test with Welch's Correction (*n* = 3). Asterisks denote *p*‐values: ^****^ *p* < 0.0001, ^***^ *p* < 0.001, ^**^ *p* < 0.01, ^*^ *p* < 0.05.

These findings were corroborated in a rat model using firefly luciferase (FFLuc) mRNA‐based LNPs, where DEPE formulations showed significantly higher bioluminescence at 0.03 mg kg^−1^ mRNA dose compared to other helper lipids for both DKP‐6 (**Figure** **5**a) and GA‐16 (Figure 5b). Composition‐3 showed higher expression in rats, and GA‐16 formulations consistently outperformed DKP‐6 counterparts. Importantly, conventional process‐4 demonstrated comparable efficacy to other processes for both DKP‐6 (Figure S3, Supporting Information) and GA‐16 (Figure S4, Supporting Information) when formulated with DEPE. Consequently, the GA‐16/DEPE formulation using composition‐3 and process‐4 was selected as the lead candidate for subsequent dose‐ranging and repeat‐dosing studies in rats. However, before initiating these studies, a comprehensive biophysical characterization was performed to elucidate how ionizable lipids, helper lipids, and LNP composition contributed to the higher protein expression observed with this optimized formulation.

### 2.5 Mechanistic Insights into Enhanced Protein Expression

#### 2.5.1 Influence of LNP Structure and Surface Properties

Cryo‐transmission electron microscopy (Cryo‐TEM) imaging revealed a correlation between LNP morphology and protein expression levels (Figure 4). LNPs with lower hOTC expression displayed heterogeneous morphology, including bleb‐like, uni‐lamellar, and core/multi‐lamellar structures. In contrast, high‐potency LNPs consistently showed homogeneous core/multi‐lamellar structures. For MC‐3 formulations (Figure 4a), DSPC‐containing LNPs showed a mix of bleb‐like, uni‐lamellar, and core/multi‐lamellar structures, corresponding to the lowest expression. DOPE, DOPC, and DEPE formulations, associated with higher expression, exhibited predominantly core/multi‐lamellar structures. Similar trends were observed for DKP‐6 (Figure 4b) and GA‐16 (Figure 4c) formulations, where DEPE‐containing LNPs consistently displayed homogeneous core/multi‐lamellar structures correlating with superior expression. Our previous work using sucrose density gradient analysis corroborated these findings, demonstrating that homogeneous core/multi‐lamellar structures are optimal for protein expression.^[^ ^49^ ^]^ Notably, while helper lipid composition primarily dictated LNP structure, overall protein expression was also influenced by LNP composition. For instance, DEPE‐based DKP‐6 LNPs maintained core/multi‐lamellar structures across all compositions, yet composition‐3 yielded higher hOTC expression (Figure 4b). This observation suggested that additional mechanisms beyond structural morphology contribute to enhanced protein expression.

**Figure 6:**
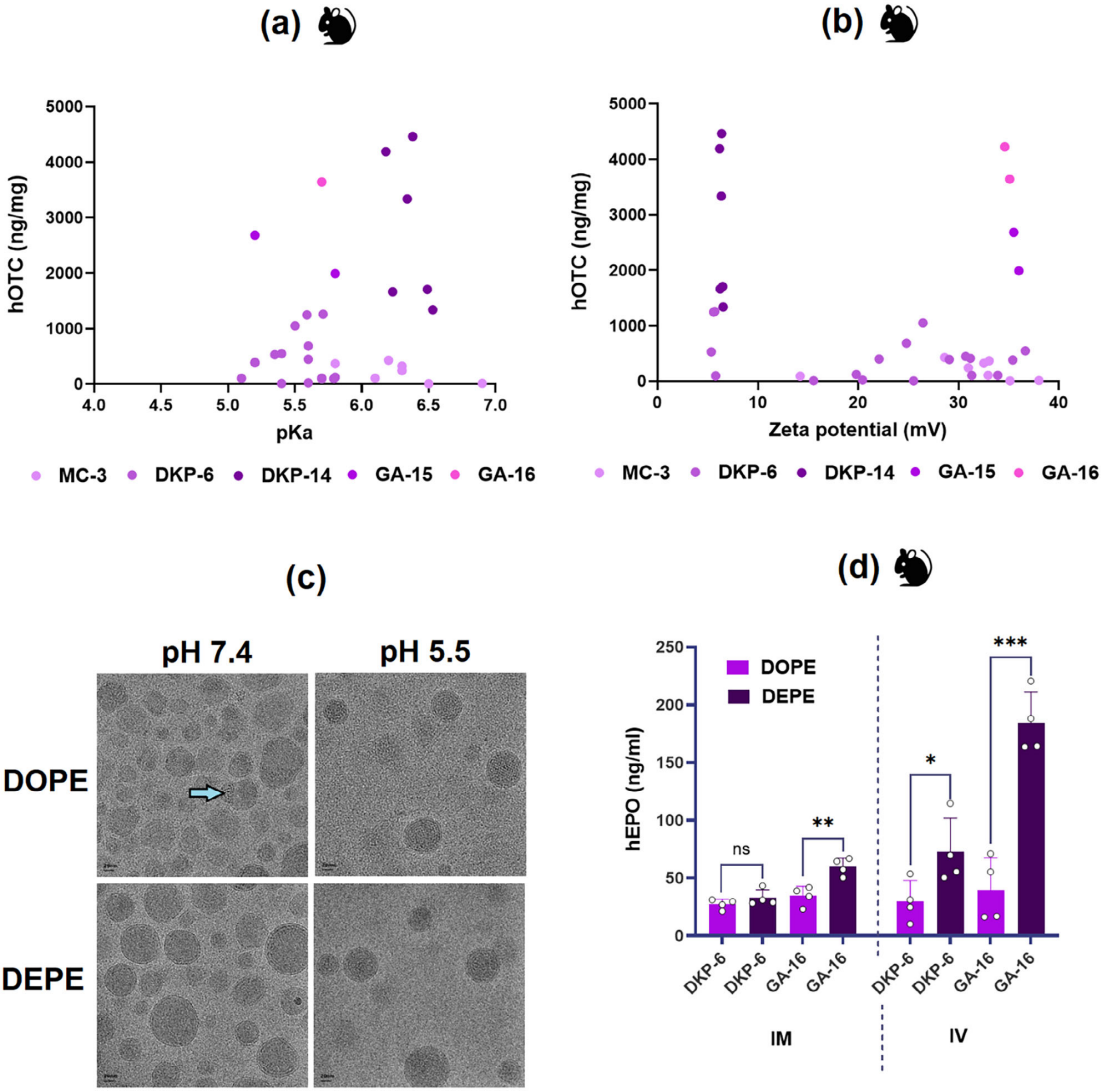
Mechanistic Insights into Enhanced Protein Expression: Influence of LNP Structure and Surface Properties. No clear correlation was observed between hOTC expression and a) surface pKa or b) zeta potential of LNPs formulated with various ionizable lipids, processes, compositions, and helper lipids. c) Cryo‐TEM images of thionine‐stained GA‐16 LNPs formulated with DOPE and DEPE at physiological pH (7.4) and endosomal pH (5.5). Structural differences and variable mRNA distribution between DOPE‐ and DEPE‐based LNPs were observed only at pH 7.4, while no significant differences were detected at pH 5.5. d) hEPO expression following intramuscular (IM) (at 0.1 µg/Animal mRNA dose) and intravenous (IV) (at 0.01 mg kg^−1^ mRNA dose) administration of DOPE‐ and DEPE‐based LNPs containing lead ionizable lipids DKP‐6 and GA‐16 in mice. The higher expression of DEPE‐based LNPs was more pronounced via the IV route, suggesting that liver tropism plays a more significant role than endosomal escape in enhancing protein expression. Student's *t*‐Test with Welch's Correction (*n* = 4). Asterisks denote *p*‐values: ^****^ *p* < 0.0001, ^***^ *p* < 0.001, ^**^ *p* < 0.01, ^*^ *p* < 0.05. ns denotes statistical insignificance.

Further biophysical characterization of surface charge revealed no discernible correlation between protein expression and either surface pKa (range: 5–7) or zeta potential (range: 5–40 mV) of the LNPs (**Figure** **6**a,b). To elucidate the impact of endosomal pH on LNP structure, thionine‐stained cryo‐TEM analysis of DOPE and DEPE‐based GA‐16 LNPs was conducted at physiological (7.4) and endosomal (5.5) pH. Structural differences and mRNA distribution variations were evident only at pH 7.4, with DOPE LNPs exhibiting bleb‐like structures and heterogeneous mRNA distribution. At pH 5.5, both DOPE and DEPE LNPs displayed similar core/multi‐lamellar structures with homogeneous mRNA distribution (Figure 6c). These findings suggest that serum protein binding‐mediated biodistribution of morphologically distinct LNPs in circulation, rather than the endosomal escape, may be the primary driver of enhanced protein expression in the liver. This hypothesis was further supported by comparing intramuscular and intravenous administration of DOPE and DEPE‐based LNPs containing DKP‐6 and GA‐16 (Figure 6d). Local delivery via intramuscular administration showed minimal differences between DOPE and DEPE formulations. In contrast, intravenous administration resulted in significantly higher human erythropoietin (hEPO) expression for DEPE‐based LNPs, reinforcing the importance of liver tropism in enhancing hepatic protein expression.

#### 2.5.2 Role of ApoE Binding and LDLR‐Mediated Hepatic Biodistribution

**Figure 7:**
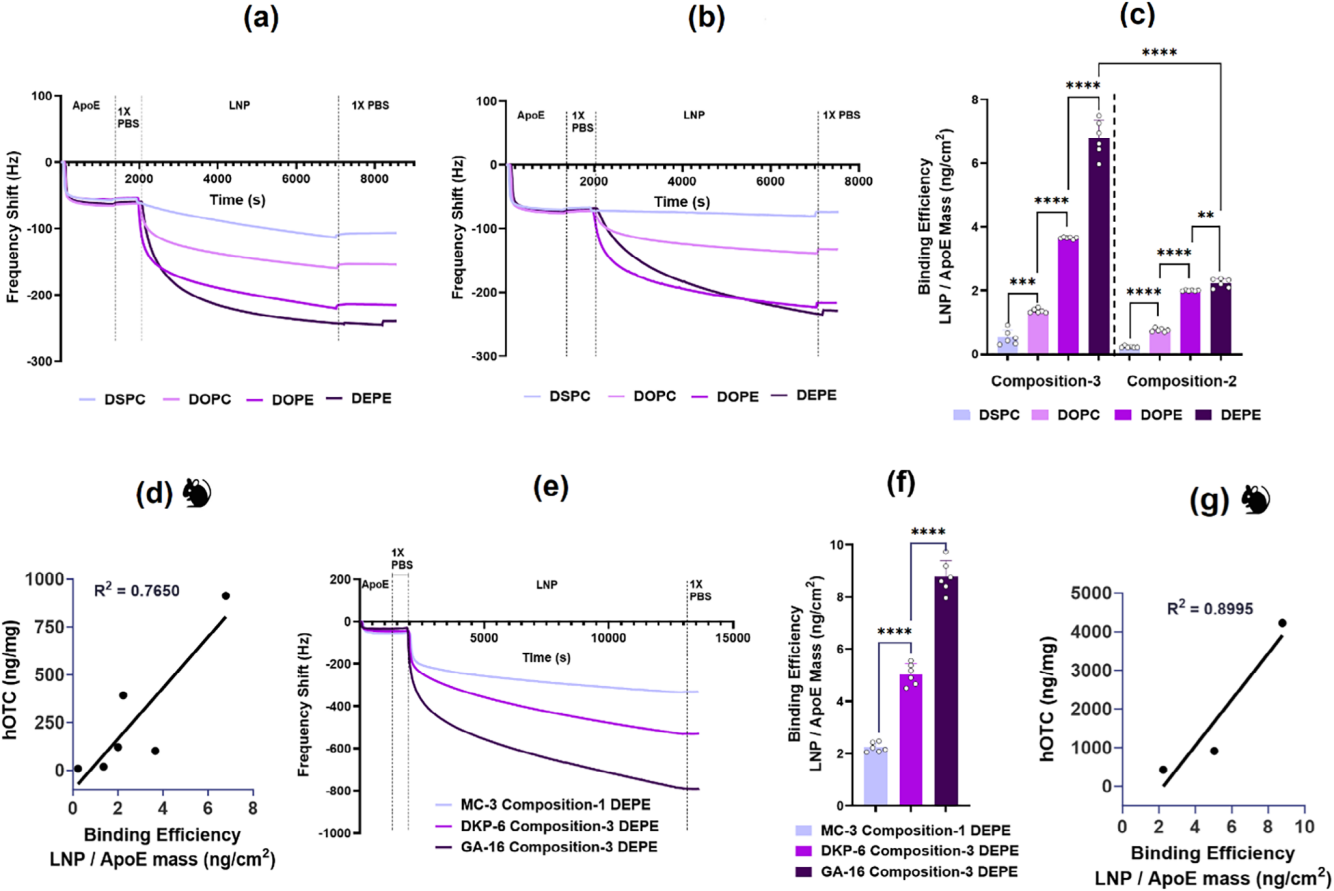
Mechanistic Insights into Enhanced Protein Expression: Role of ApoE Binding‐ In‐vitro Evaluation using QCMD. QCMD Evaluation of DKP‐6 LNPs with Various Helper Lipids: a) Time‐dependent negative frequency shift for composition‐3, b) Time‐dependent negative frequency shift for composition‐2, c) ApoE binding efficiency expressed as LNP to ApoE mass ratio, d) Correlation between ApoE binding and hOTC expression in mice. QCMD Evaluation of Lead LNPs with Different Ionizable Lipids: e) Time‐dependent negative frequency shift, f) ApoE binding efficiency expressed as LNP to ApoE mass ratio, g) Correlation between ApoE binding and hOTC expression at 1 mg kg^−1^ mRNA dose in mice. Overall, ApoE binding efficiency followed hierarchical trends as follows for helper lipids: DEPE >DOPE >DOPC >DSPC, Compositions: composition‐3 >composition‐2, and ionizable lipids: GA‐16 >DKP‐6 >MC‐3. A strong positive correlation was demonstrated between LNP ApoE binding efficiency and hOTC expression levels. Student's *t*‐Test with Welch's Correction (*n* = 6). Asterisks denote *p*‐values: ^****^ *p* < 0.0001, ^***^ *p* < 0.001, ^**^ *p* < 0.01, ^*^ *p* < 0.05.

ApoE binding affinity of LNPs was assessed using quartz crystal microbalance with dissipation monitoring (QCM‐D), where a negative shift in frequency indicates adsorption of any material. As shown in **Figure** **7**a, DKP‐6 LNPs formulated with lead composition‐3 exhibited ApoE binding in the order: DEPE >DOPE >DOPC >DSPC, based on the magnitude of frequency shift. A similar trend (DEPE ≥ DOPE >DOPC >DSPC) was observed for LNPs formulated with composition‐2 (Figure 7b). ApoE binding efficiency, determined by LNP‐to‐ApoE mass ratio, mirrored this order across both compositions (Figure 7c), indicating that DEPE‐based LNPs exhibit the strongest ApoE interaction. Notably, hepatic expression of hOTC correlated with ApoE binding. As shown in Figure 7d, hOTC expression in the liver following administration of DKP‐6 LNPs formulated with compositions‐2 and ‐3 and the four different helper lipids, was strongly correlated with ApoE binding efficiency (*R* ^2^ = 0.7650). Similarly, LNPs formulated with different ionizable lipids showed varying ApoE binding and liver expression profiles. Frequency shifts (Figure 7e) and LNP‐to‐ApoE mass ratios (Figure 7f) ranked GA‐16 >DKP‐6 >MC‐3, consistent with ApoE adsorption efficiency. Liver expression of hOTC mirrored this trend, showing a strong linear correlation with ApoE binding (R^2^ = 0.8995), suggesting that hepatic protein expression is highly dependent on ApoE‐mediated transport across ionizable and helper lipid combinations (Figure 7g).

**Figure 8:**
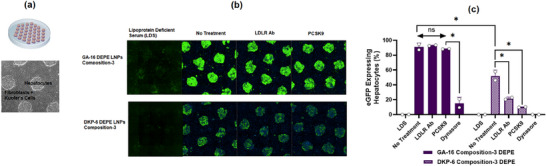
Mechanistic Insights into Enhanced Protein Expression: Role of LDLR‐Mediated Uptake‐ In‐vitro Evaluation in HEPATOMUNE Model. a) Schematic of the HEPATOMUNE tri‐culture model comprising hepatocytes, fibroblasts, and Kupffer cells, designed to mimic the physiological microenvironment of the liver. b) Microscopic images and c) quantification of eGFP‐expressing hepatocytes in HEPATOMUNE tri‐cultures transfected with lead LNPs of GA‐16 and DKP‐6 under various treatment conditions. Treatments included lipoprotein‐deficient medium (lacking ApoE), LDLR antibody and PCSK9 (inhibiting LDLR receptor function), and Dynasore (inhibiting dynamin GTPase activity required for clathrin‐dependent coated‐vesicle formation). Results demonstrated that while uptake of both GA‐16 and DKP‐6‐based LNPs was dependent on ApoE and clathrin‐coated vesicle formation, only DKP‐6‐based LNPs required LDLR for efficient cellular entry. Student's *t*‐Test with Welch's Correction (Twenty‐five fields per well were captured across three independent experiments conducted in duplicate). Asterisks denote *p*‐values: ^****^ *p* < 0.0001, ^***^ *p* < 0.001, ^**^ *p* < 0.01, ^*^ *p* < 0.05. ns denotes statistical insignificance.

Cellular uptake mechanisms were further investigated using the HEPATOMUNE tri‐culture model, comprising primary hepatocytes, stromal cells, and Kupffer cells, which replicates the physiological hepatic microenvironment (**Figure** **8**a). Enhanced green fluorescence protein (eGFP) expression in primary hepatocytes was observed with GA‐16 LNPs compared to DKP‐6 LNPs (Figure 8b). Both formulations showed reduced uptake in lipoprotein‐deficient serum (LDS), while normal ApoE‐containing medium restored expression, confirming ApoE dependence for hepatocyte uptake. Interestingly, LDLR blockade via antibody or proprotein convertase subtilisin/kexin type 9 (PCSK9) treatment significantly reduced both eGFP expression (Figure 8b) and the percentage of eGFP‐expressing cells (Figure 8c) for DKP‐6 LNPs but had no effect on GA‐16 LNPs, indicating that DKP‐6 strongly relies on LDLR‐mediated uptake, whereas GA‐16 utilizes an alternative pathway. On the other hand, a significant reduction in eGFP‐expressing cells observed with both formulations upon treatment with Dynasore, a specific dynamin guanosine triphosphatase (GTPase) inhibitor, indicated that these LNPs primarily utilize clathrin‐dependent endocytic pathways for cellular internalization (Figure 8c).

**Figure 9:**
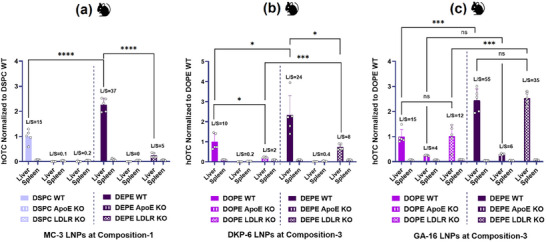
Mechanistic Insights into Enhanced Protein Expression: Role of ApoE Binding and LDLR‐Mediated Hepatic Biodistribution In‐Vivo. Various helper lipid‐based LNPs of three different ionizable lipids: a) MC‐3, b) DKP‐6, c) GA‐16 were administered to WT, ApoE KO, and LDLR KO mice at 1 mg kg^−1^ mRNA dose. Each ionizable lipid was formulated using its corresponding lead composition. Across all three ionizable lipids, DEPE‐based LNPs demonstrated superior expression compared to DSPC‐ and DOPE‐based formulations. While liver protein expression was strongly dependent on ApoE‐mediated transport, it showed relatively less dependence on LDLR‐mediated uptake. Notably, GA‐16‐based LNPs exhibited equivalent hOTC expression in both WT and LDLR KO mice, indicating independence from LDLR‐mediated uptake pathways. These in vivo findings correlated well with results from in vitro evaluations conducted in the human HEPATOMUNE model. Student's *t*‐Test with Welch's Correction (*n* = 5). Asterisks denote *p*‐values: ^****^ *p* < 0.0001, ^***^ *p* < 0.001, ^**^ *p* < 0.01, ^*^ *p* < 0.05. ns denotes statistical insignificance.

These in vitro findings were corroborated with in vivo studies using WT, ApoE KO, and LDLR KO mice administered with LNPs formulated using DEPE, DOPE, or DSPC with MC‐3, DKP‐6, or GA‐16 (**Figure** **9**). All formulations demonstrated liver‐specific expression with no significant splenic expression across models. In general, hOTC expression was strongly dependent on ApoE‐mediated transport and, to a lesser extent, on LDLR‐mediated uptake, depending on the ionizable lipid used. In WT mice, MC‐3 LNPs formulated with DEPE yielded significantly higher hOTC levels than those with DSPC, while no expression was observed in ApoE KO mice (Figure 9a), confirming reliance on ApoE. DEPE‐based MC‐3 LNPs showed marginal expression in LDLR KO mice, whereas DSPC‐based LNPs showed no expression, indicating greater ApoE‐mediated transport for DEPE formulations. Similarly, DKP‐6 LNPs with DEPE outperformed DOPE‐based formulations in WT mice (Figure 9b), and neither showed liver expression in ApoE KO mice. Similarly, in LDLR KO mice, DEPE‐based DKP‐6 LNPs yielded higher expression than DOPE‐based LNPs, suggesting enhanced ApoE‐mediated delivery by DEPE. GA‐16 LNPs followed the same trend, with DEPE‐based formulations outperforming DOPE in WT mice (Figure 9c). Notably, both DOPE and DEPE‐based LNPs of GA‐16 demonstrated equivalent expression in ApoE KO mice, although expression levels were lower compared to their respective LNPs in WT mice, distinguishing them from other ionizable lipids. These findings definitively established that DEPE‐based LNPs exhibit enhanced ApoE binding, resulting in increased hepatic transport compared to their DOPE‐based counterparts. A remarkable observation was that GA‐16‐based LNPs maintained protein expression in the liver independently of LDLR‐mediated uptake, regardless of the helper lipid employed. This was evidenced by equivalent hOTC expression levels in both LDLR KO and WT mice for both DOPE and DEPE‐based GA‐16 LNPs. Consistent with the pattern observed in WT mice, DEPE‐based LNPs of GA‐16 achieved higher hOTC expression compared to DOPE‐based LNPs in LDLR KO mice. These observations further substantiated the superior ApoE binding capability of DEPE‐based LNPs, explaining their enhanced liver targeting efficiency relative to DOPE‐based LNPs. Figures 9a and 8b,c illustrate that DEPE‐based LNPs consistently achieved approximately twice the relative hOTC expression compared to LNPs formulated with either DSPC or DOPE across all mouse models, except for ApoE KO mice. This observation reinforces the superior ApoE binding capacity of DEPE‐based LNPs, regardless of the ionizable lipid used. In LDLR KO mice, the relative hOTC expression followed a descending order among ionizable lipids: GA‐16, DKP‐6, and MC‐3, with GA‐16‐based LNPs demonstrating near independence from LDLR‐mediated uptake. These findings align closely with the in vitro evaluations conducted using human HEPATOMUNE cells, where GA‐16‐based LNPs showed no reliance on LDLR‐mediated uptake. In contrast, DKP‐6‐based LNPs exhibited a significant reduction in eGFP‐expressing cells when LDLR receptors were blocked, as evidenced in Figure 8b,c, further corroborating the distinct uptake mechanisms of these formulations.

### 2.6 Lead GA Formulation

#### 2.6.1 Achievement of Key Preclinical Milestones with Successful Dose–Ranging and Repeat Administration in Rats

**Figure 10:**
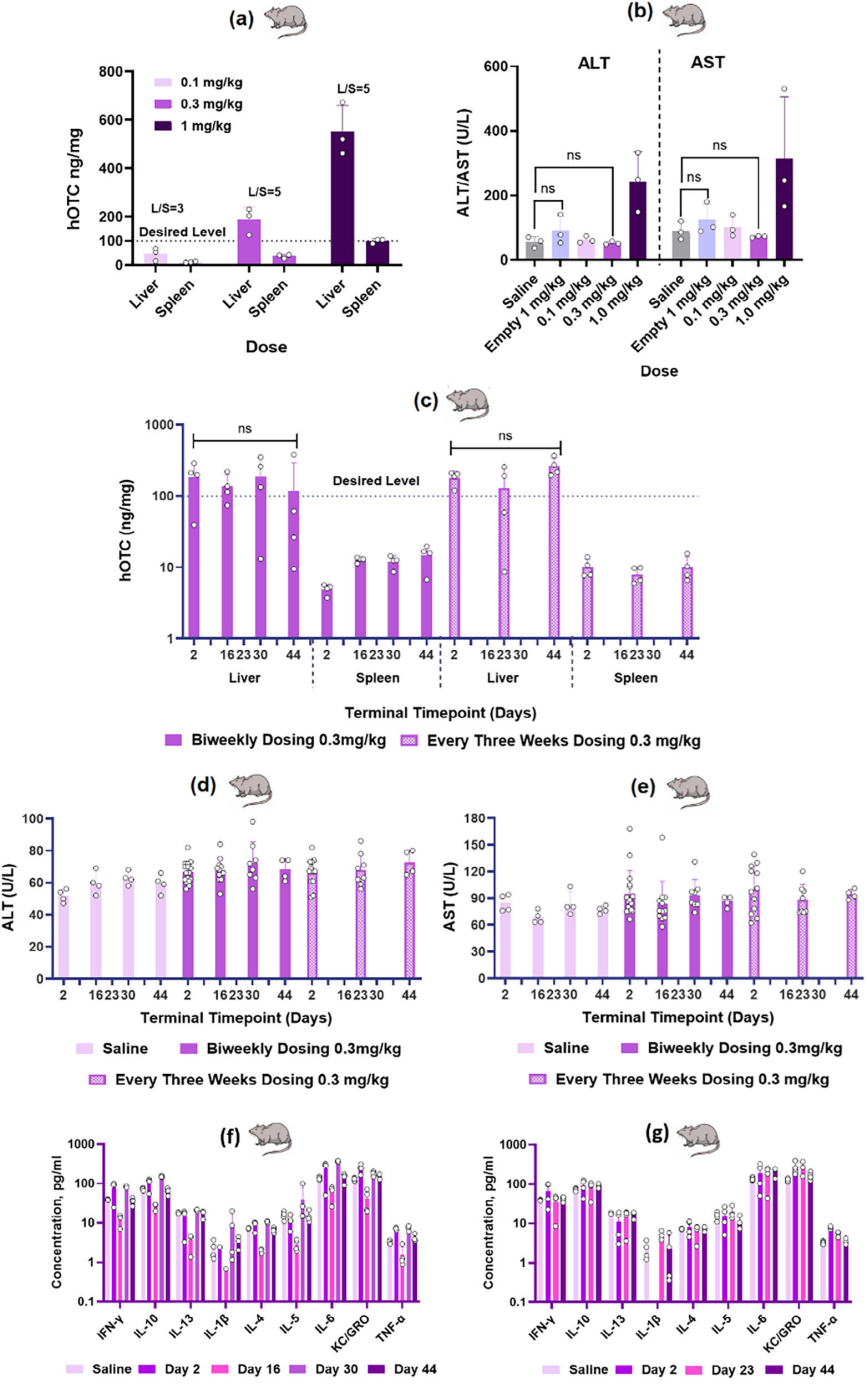
Lead GA Formulation: Achievement of Key Preclinical Milestones with Successful Dose–Ranging and Repeat Administration in Rats. Evaluation of LNP formulation containing lead ionizable lipid GA‐16 with DEPE helper lipid, formulated using composition‐3 and process‐4, in rats. a) Dose‐dependent increase in hOTC expression, with desired levels exceeding 100 ng mg^−1^ achieved at doses of 0.3 mg kg^−1^ and above. b) No elevation in liver enzymes ALT and AST at 0.3 mg kg^−1^ dose, indicating desired tolerability. c) No significant difference in desired hOTC expression levels with both biweekly and every‐three‐weeks dosing regimens, confirming sustained protein expression during repeat dosing. No significant increase in d) ALT, e) AST, or cytokine levels for f) biweekly and g) every three weeks dosing regimens, demonstrating a favorable tolerability profile for this lead LNP formulation. Student's *t*‐Test with Welch's Correction (*n* = 3) for (b) and One Way ANOVA with Welch's Correction (*n* = 4) for (c). ns denotes statistical insignificance.

The lead candidate selected for dose ranging and repeat dosing studies in rats was the LNP formulation combining GA‐16 ionizable lipid with DEPE, prepared using composition‐3 and process‐4. Comprehensive dose‐response evaluation was conducted across three dose levels: 0.1, 0.3, and 1 mg kg^−1^ (**Figure** **10**a). The 0.3 mg kg^−1^ dose successfully achieved the target hOTC expression threshold of 100 ng mg^−1^ while maintaining favorable liver tolerability profiles. Hepatic biomarker analysis demonstrated that ALT/AST levels at this dose remained comparable to saline‐treated controls (Figure 10b), indicating a favorable tolerability profile. Although the 1 mg kg^−1^ dose yielded significantly higher hOTC expression than required (Figure 10a), it was accompanied by elevated ALT/AST levels compared to saline controls (Figure 10b). Notably, empty LNPs (without mRNA) demonstrated excellent tolerability even at lipid concentrations equivalent to the 1 mg kg^−1^ mRNA dose, with ALT/AST levels remaining comparable to saline controls, indicating an inherently favorable safety profile of the delivery system. Based on these findings, the 0.3 mg kg^−1^ dose was selected for repeat dosing studies to maintain an optimal balance between efficacy and tolerability.

The repeat dosing evaluation employed two distinct regimens: biweekly and every three weeks administration at 0.3 mg kg^−1^. Both dosing schedules successfully maintained desired hOTC expression levels (Figure 10c), demonstrating consistent protein expression without significant reduction upon repeated administration. The robust tolerability profile of this formulation was further confirmed by stable ALT (Figure 10d), AST (Figure 10e), and cytokine (Figure 10f,g) levels across both dosing regimens, with no significant elevations observed. While direct assessment of ammonia metabolism activity was not possible due to the unavailability of hOTC KO rat models, previous studies with the DKP family lead formulation in mice (Figure 2a–c) suggest that the hOTC levels exceeding 100 ng mg^−1^ should effectively maintain metabolic activity for 2–3 weeks. This extrapolation provides reasonable confidence in the therapeutic potential of the dosing regimens established for the GA‐16/DEPE formulation.

## 3 Conclusion

This work demonstrates that helper lipids play a crucial role in determining ApoE binding and liver tropism of LNPs, while ionizable lipids influence both ApoE binding and LDLR‐mediated uptake. These insights are particularly valuable for advancing the development of LNP formulations across multiple therapeutic applications. Beyond mRNA‐based protein replacement therapy, these findings have significant implications for various gene therapy and gene editing approaches targeting rare diseases. Of particular interest is the application in disorders like familial hypercholesterolemia, which is characterized by severe or complete LDLR deficiency. In such cases, the strategic formulation of LNPs becomes critical for therapeutic success. By incorporating higher ApoE‐binding helper lipids such as DEPE, combined with specialized ionizable lipids like GA‐16 that can function independently of LDLR‐mediated uptake, liver biodistribution and cellular uptake can be accomplished even in the absence of LDLR. This innovative approach enables achieving therapeutically relevant levels of cargo delivery at lower and well‐tolerated doses, thereby improving the safety profile of the treatment. The ability to fine‐tune LNP compositions based on these mechanistic insights opens new possibilities for treating LDLR‐deficient conditions and potentially other liver‐targeted therapies. This understanding of LNP‐serum protein interactions and cellular uptake mechanisms represents a significant advancement in the field of nanomedicine, particularly for developing more effective therapeutic strategies for genetic disorders affecting metabolism in liver.

## 4 Experimental Section

### Materials

FFLuc mRNA, eGFP mRNA, hOTC mRNA, hEPO mRNA, and novel ionizable lipids were synthesized internally at Sanofi. DMG‐PEG‐2000, DOPE, and DEPE were obtained from NOF America Corporation (White Plains, NY, USA). Benchmark ionizable lipid MC‐3, and helper lipids–DSPC and DOPC were purchased from Avanti Polar Lipids (Alabaster, AL, USA). Cholesterol was sourced from Millipore Sigma (Burlington, MA, USA), and ethanol was acquired from Fisher Scientific. Citrate buffer (pH 4.5) and sodium chloride solution were purchased from Boston Scientific (Marlborough, MA, USA). The structures of benchmark and lead novel ionizable lipids and helper lipids are shown in Figure S5 (Supporting Information). Detailed synthesis procedure for the lead novel ionizable lipids DKP‐6 and GA‐16, along with their 1H NMR spectra, has been added as Figures S6 and S7 (Supporting Information), respectively. Human ApoE ≥ 95% (SDS‐PAGE) was purchased from Sigma.

### Preparation of LNP Formulations

The schematics of the processes used for the preparation of mRNA LNP formulations are presented in Figure S1 (Supporting Information). Process‐4, described previously,^[^ ^50^ ^]^ involved the rapid mixing of an ethanolic lipid mixture with an aqueous buffered mRNA solution at acidic pH, using a fixed lipid‐to‐mRNA ratio under controlled conditions to produce a uniform dispersion of LNPs. The resulting dispersion was subjected to ultrafiltration and diafiltration into a suitable diluent, diluted to the final concentration, sterile‐filtered, and stored at −80 °C until use. mRNA LNP formulations prepared using Process‐1, −2, and −3 followed modified protocols.^[^ ^51^ ^]^ Briefly, empty LNPs (without mRNA) were first prepared using Process‐4, heated to 65 °C, and then mixed with an aqueous mRNA solution at a fixed lipid‐to‐mRNA ratio. The mixture was incubated at 65 °C for 15 min, cooled in an ice bath, and processed by ultrafiltration and diafiltration into an appropriate diluent. Final formulations were adjusted to the desired concentration, filtered, and stored at −80 °C. While Process‐1 employed instantaneous mixing of mRNA with empty LNPs, Processes 2 and 3 differed in the kinetics of mRNA incorporation. Specifically, in Process‐2, the empty LNPs were gradually mixed with the mRNA solution using a decreasing lipid‐to‐mRNA ratio, whereas in Process‐3, the ratio was increased stepwise to reach the final fixed ratio. All other steps remained consistent across the three methods. The molar compositions evaluated for ionizable lipid screening and formulation optimization are detailed in Table 1. Physicochemical characteristics of the selected lead and control LNP formulations are summarized in Table S1 (Supporting Information).

### LNP Characterization

mRNA encapsulation efficiency and concentration were determined using the RiboGreen assay (Quant‐it Ribogreen Assay Kit, Thermo Fisher Scientific) following the manufacturer's protocol. LNPs were characterized using dynamic light scattering (DLS) on a Malvern Zetasizer instrument to measure Z‐average diameter, polydispersity index (PDI), and zeta potential. Surface pKa of the LNPs was evaluated using the 2‐(p‐toluidinyl)naphthalene‐6‐sulphonic acid (TNS) assay. For this analysis, LNPs were diluted in a series of pH‐specific buffers ranging from pH 3.0 to 9.0 at 0.5 pH unit intervals and mixed with TNS dye at a final concentration of 6 µM per well. Fluorescence measurements were recorded at excitation wavelength 322 nm and emission wavelength 431 nm. The surface pKa value was determined as the pH at which 50% protonation of the ionizable amine groups occurred, calculated from the fluorescence pH titration curves generated for each LNP formulation.

### Cryo‐TEM Imaging of the LNPs

Samples were prepared by plunge‐freezing vitrification and imaged using brightfield cryo‐TEM techniques. For contrast enhancement, selected samples were stained with 0.5 mM thionine acetate high‐purity biological stain, according to previously established methods.^[^ ^52^ ^]^ Quantifoil TEM grids (Quantifoil GmbH) were subjected to glow‐discharge treatment for 25 s in rarefied room air using a Pelco EasiGlow system (Ted Pella). Sample vitrification was performed in a Vitrobot Mk IV chamber (ThermoFisher) maintained at 22 °C and 95% relative humidity, where samples were applied to grids, blotted, and rapidly plunged into liquid ethane. Each LNP formulation was vitrified on Quantifoil R2/1 200 mesh grids with and without a continuous carbon film (2 nm nominal thickness) to optimize visualization conditions. Imaging was conducted on a Talos Arctica transmission electron microscope (FEI Company) equipped with a K2 direct electron detector (Gatan) and controlled by Serial EM software. Micrographs were acquired at nominal magnifications of 49 000× and 130 000×. For each specimen, both grid types were systematically screened, and images were collected from grids exhibiting optimal ice thickness and minimal contamination to ensure representative visualization of the LNP structures.

### QCM‐D Evaluation

Measurements were performed using Au‐coated sensors (QSX301, Biolin Scientific) with a 4.95 MHz resonance frequency. Sensor preparation involved sequential cleaning steps: 10 min UV‐Ozone treatment, 5 min base piranha treatment, thorough rinsing with MilliQ water, N2 drying, and a final 10 min UV‐Ozone treatment. An E4 QCM‐D instrument (Q‐Sense Inc., Gothenburg, Sweden) was used to monitor changes in resonance frequency (ΔF) and dissipation (ΔD) for odd overtones, with all measurements referenced to PBS buffer‐equilibrated baseline values. Solution delivery was controlled using a peristaltic pump operating at 40 µL min^−1^. ApoE adsorption was initiated by introducing 0.1 mg mL^−1^ ApoE in PBS over the sensors.^[^ ^53^ ^]^ Following 20 min of continuous flow, an intermittent pumping protocol was implemented (50 µL continuous flow followed by 4–9 min pause) to conserve material. After 80 min of adsorption, the ApoE layers were equilibrated with PBS buffer (40 µL min^−1^, ≈20 min). LNP‐ApoE interactions were investigated by introducing 0.05 mg mL^−1^ LNP in PBS over the equilibrated ApoE layer. The flow protocol consisted of 10 min continuous pumping followed by intermittent delivery (50 µL over 1 min, followed by 4 min pause) for 80 min total interaction time. The system was then rinsed with PBS buffer under continuous flow conditions.

### Cellular Uptake Study Using Human HEPATOMUNE Model

In vitro studies were conducted using the Human HEPATOMUNE model (BioIVT, Baltimore, MD) in 96‐well format, following previously established protocols.^[^ ^54^, ^55^ ^]^ The culture medium was replaced with supplemented Williams' E Medium (WEM) containing GlutaMax, insulin‐transferrin‐selenium, and ascorbic acid (0.05 mg mL^−1^). For receptor‐blocking studies, cells were pre‐treated with either LDLR‐neutralizing antibody, recombinant human PCSK9, or dynamin inhibitor I (dynasore). LNPs encapsulating eGFP mRNA were pre‐incubated for 30 min with either human ApoE or 20% lipoprotein‐deficient calf serum in supplemented media. These preparations were added to pre‐treated cells at a 1:1 ratio (100 µL total volume per well). Cells were exposed to LNPs at concentrations ranging from 0.5 to 2 µg mL^−1^ and incubated for 4 h at 37 °C. Following incubation, the medium was replaced with HEPATOMUNE maintenance medium for subsequent live imaging analysis. Time‐lapse imaging was performed using an Operetta high‐content microscope (Revvity) equipped with a 20× water immersion objective, maintaining physiological conditions (37 °C, 10% CO2). Twenty‐five fields per well were captured across three independent experiments conducted in duplicate. Image analysis was performed using Harmony software (Revvity), where individual field images were consolidated into global images. DAPI staining enabled automated hepatocyte island identification and exclusion of non‐hepatocyte cells. Cell masks were applied for hepatocyte area detection, and eGFP fluorescence intensity was quantified on a per‐hepatocyte basis.

### Animal Studies

Multiple mouse strains were utilized: CD‐1 males (6–8 weeks, Charles River Laboratories) for ionizable lipid screening, formulation optimization, and dose‐ranging studies; C57BL/6 and hemizygous OTC^spf‐ash^ males (4–8 weeks, Jackson Laboratories) for ammonia challenge studies; and C57BL/6 WT, ApoE KO, and LDLR KO males (6–8 weeks, Jackson Laboratories) for ApoE and LDLR‐mediated tropism studies. Male SD rats were employed for dose range and repeat dose studies. Animals were maintained in pathogen‐free conditions with a 12 h light/dark cycle and provided ad libitum access to food and water. Test materials were administered as single intravenous doses (5 mL kg^−1^) via tail vein on Day 1. Animals were euthanized 24 h post‐dose using carbon dioxide asphyxiation followed by thoracotomy. Terminal blood collection was performed via cardiac puncture using serum separator tubes. For ammonia challenge studies (Days 15 and 22), fasted animals received intraperitoneal injections of freshly prepared 0.2 m ammonium chloride solution (25 mL kg^−1^). Blood samples were collected via the tail vein 30 min post‐ammonium chloride administration. Serum was extracted from whole blood and stored at −70 °C for subsequent ALT/AST analysis by ELISA. Following euthanasia, the whole liver and spleen were harvested, with 3–6 liver biopsies (4 or 8 mm punches) collected per animal. Tissue samples were snap‐frozen in liquid nitrogen and stored at −70 °C for later hOTC quantification by ELISA.

### Ethics Statement

All animal studies were conducted at Alpha Preclinical (North Grafton, MA) in accordance with US National Institutes of Health guidelines and relevant local, state, and federal regulations. Experimental protocols were approved by Tufts University's Institutional Animal Care and Use Committee (IACUC) and adhered to ethical guidelines for animal research.

### Statistical Analysis

GraphPad PRISM was used to perform statistical analysis and plot the graphs. Comparisons between the means (± SD) shown in the bar plots were performed using either Student's *t*‐Test with Welch's correction or one‐way ANOVA with Welch's correction.

## Conflict of Interest

The authors declare no conflict of interest.

## Author Contributions

The manuscript was written through the contributions of all authors. All authors have given approval to the final version of the manuscript. A.S. performed conceptualization, methodology, investigation, visualization, and wrote, reviewed, and edited the final manuscript. C.O., T.D., N.V.‐M., P.P., N.K., S.M., R.L., J.S., L.B., H.W., D.B.P., and B.Y. performed experiments. A.D. and H.D. reviewed and edited the manuscript. S.K. performed conceptualization, methodology, supervision, project administration, wrote, reviewed, and edited the final manuscript. F.D.R. performed project administration and wrote, reviewed, and edited the final manuscript.

## Supporting information



Supporting Information

## Data Availability

The data that support the findings of this study are available from the corresponding author upon reasonable request.
