# Supplementary material for: Potent Liver‐Tropic mRNA Lipid Nanoparticles: ApoE‐Mediated Delivery Through a Low‐Density Lipoprotein Receptor Independent Uptake Mechanism
Source: Adv Mater. 2025 Nov 29;38(22):e17893. doi: 10.1002/adma.202517893 (PMC13088227; doi:10.1002/adma.202517893)
Supplement: Supplementary file 1 — Supporting Information [file ADMA-38-e17893-s001.docx]

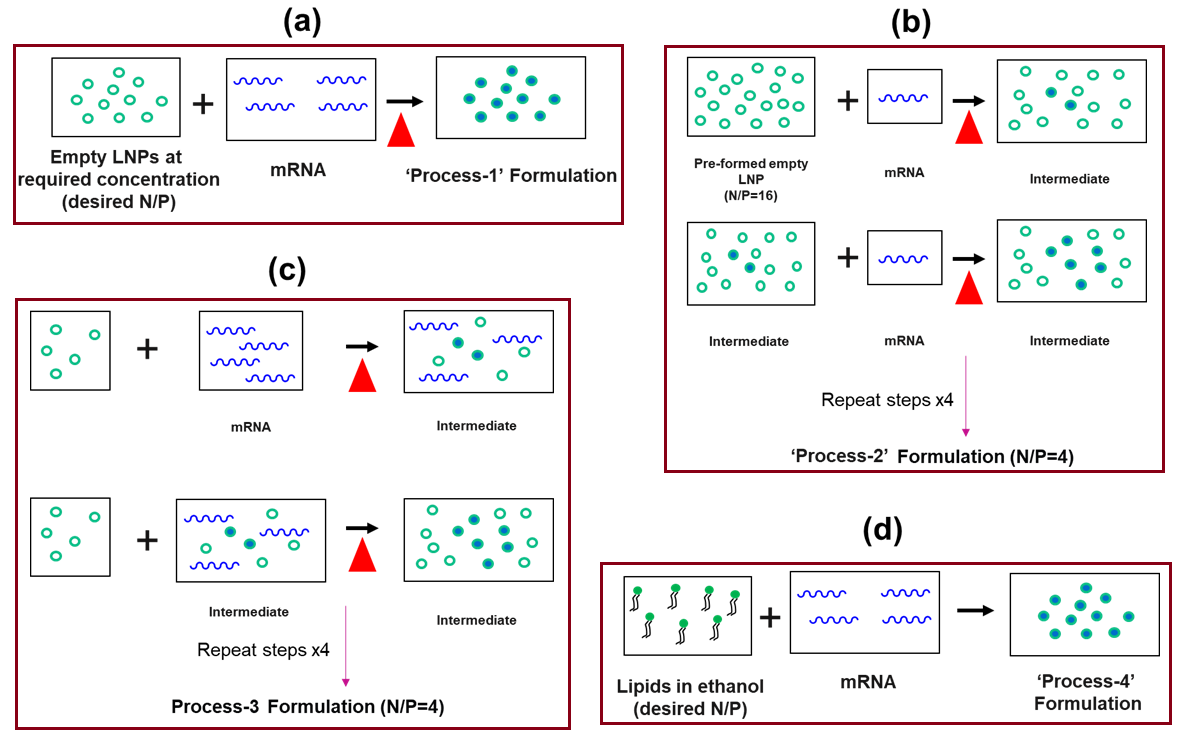


**Figure S1: Schematic Representation of LNP Formulation Processes.** (a) Process-1, (b) Process-2, (c) Process-3, and (d) Process-4. Process-4 represents the conventional LNP formulation method, where ethanol-dissolved lipids are mixed with mRNA in acidic buffer, followed by diafiltration into a suitable diluent. Empty LNPs were then formulated using process-4 without mRNA for novel Processes -1, -2, and -3 that utilized mixing of mRNA solution in water with these empty LNPs at higher temperature followed by diafiltration into a suitable diluent. Unlike instantaneous mixing used in process-1 the empty LNPs were mixed with mRNA solution gradually with decreasing and increasing lipid to mRNA ratio to reach a fixed final ratio for Process-2 and Process-3, respectively.

**Figure S2: Impact of DMG-PEG-2000 Content Reduction on LNP Performance.** Evaluation of hOTC expression at 1 mg/kg mRNA dose in mice using DKP-6 LNPs formulated with DOPE and DEPE helper lipids, comparing 3 mol% versus 2 mol% DMG-PEG-2000 content. No significant difference in protein expression was observed between the two DMG-PEG-2000 concentrations for either helper lipid-based LNP formulation. Student’s T-Test with Welch’s Correction (n = 5). ns denotes statistical insignificance.


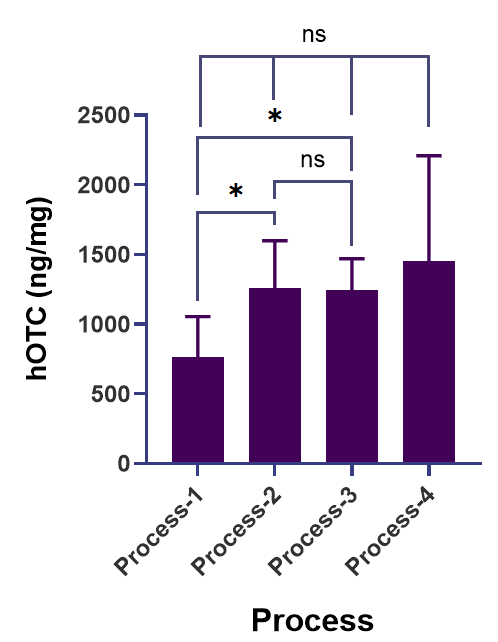


**Figure S3: Impact of Process on Protein Expression using DKP-6 LNPs containing DEPE with Composition-3.** Protein expression in the liver at 1 mg/kg mRNA dose after administering LNPs of DKP-6 with helper lipid DEPE was evaluated across formulations using process-1, -2, -3, and -4. The protein expression was higher for LNPs formulated using processes -2 and -3 compared to those using process-1, while no significant difference was observed between these processes and process-4. Student’s T-Test with Welch’s Correction (n = 5). Asterisks denote p-values: ****p < 0.0001, ***p < 0.001, **p < 0.01, *p < 0.05. ns denotes statistical insignificance.


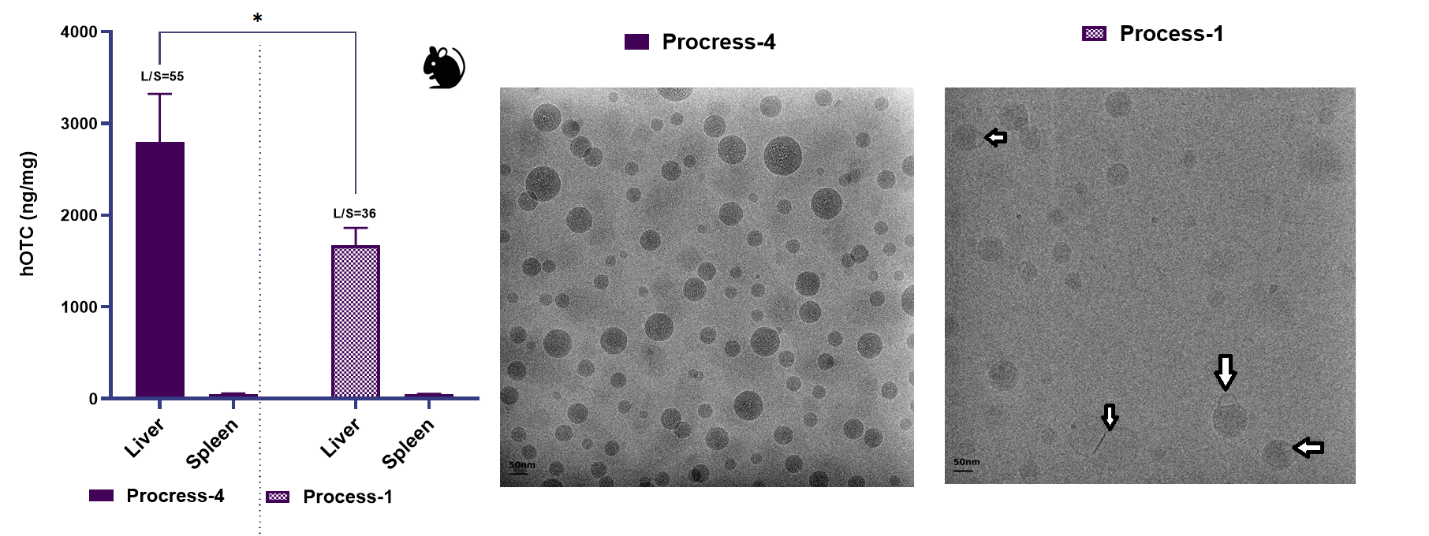


**Figure S4: Impact of Process on Tissue Distribution and LNP Structure.** Protein expression in liver and spleen at 1 mg/kg mRNA dose in mice following administration of GA-16 LNPs containing DEPE with Composition-3, formulated using process-1 and process-4, with corresponding cryo-TEM images of these formulations. LNPs formulated using process-4 demonstrated higher protein expression compared to those using process-1. This performance difference correlated with structural characteristics observed via cryo-TEM: process-4 yielded predominantly homogeneous multi-lamellar structures, whereas process-1 resulted in heterogeneous structures with blebs. Student’s T-Test with Welch’s Correction (n = 5). Asterisks denote p-values: ****p < 0.0001, ***p < 0.001, **p < 0.01, *p < 0.05.


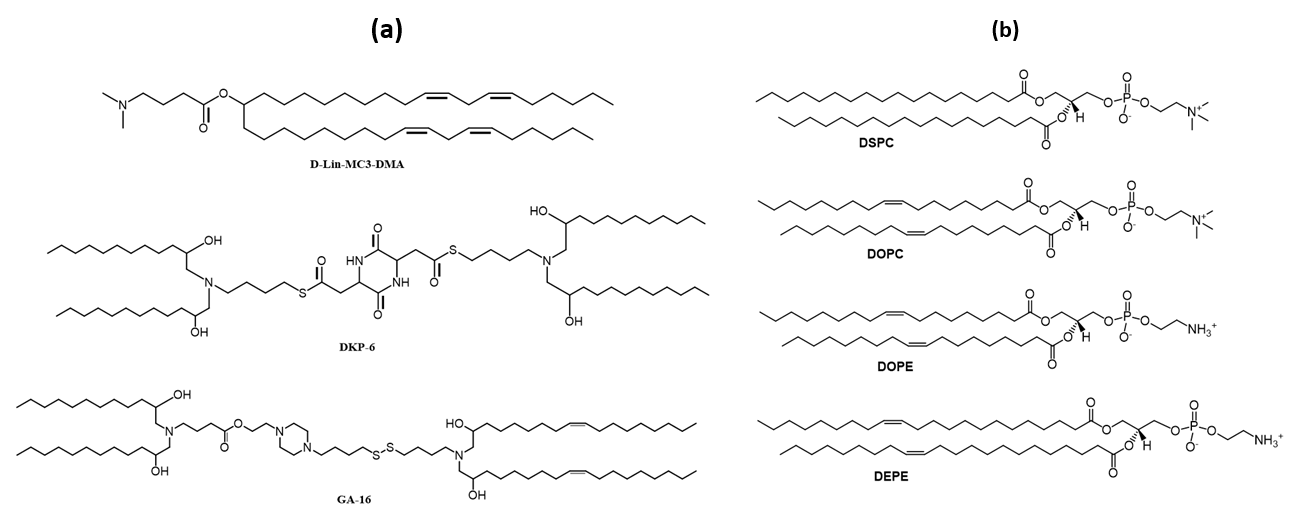


**Figure S5: Chemical Structures of Lipids.** (a) Ionizable Lipids and (b) Helper Lipids

**Table S1: Physicochemical characteristics of the selected lead and control LNP formulations**

| ***Ionizable Lipid*** | ***Helper Lipid*** | ***Composition*** | ***Process*** | ***Size (nm)*** | ***PdI*** | ***Encapsulation (%)*** |
| --- | --- | --- | --- | --- | --- | --- |
| DKP-6 | DOPE | Composition-4 | Process-1 | 111 | 0.190 | 86 |
| DKP-6 | DEPE | Composition-4 | Process-1 | 120 | 0.170 | 92 |
| DKP-6 | DOPE | Composition-4 | Process-2 | 138 | 0.184 | 93 |
| DKP-6 | DEPE | Composition-4 | Process-2 | 111 | 0.150 | 93 |
| DKP-6 | DOPE | Composition-4 | Process-3 | 124 | 0.156 | 90 |
| DKP-6 | DEPE | Composition-4 | Process-3 | 118 | 0.100 | 96 |
| DKP-6 | DOPE | Composition-4 | Process-4 | 72 | 0.143 | 94 |
| DKP-6 | DEPE | Composition-4 | Process-4 | 74 | 0.167 | 95 |
| DKP-6 | DSPC | Composition-1 | Process-4 | 128 | 0.076 | 90 |
| DKP-6 | DOPC | Composition-1 | Process-4 | 79 | 0.093 | 82 |
| DKP-6 | DEPE | Composition-1 | Process-4 | 100 | 0.076 | 91 |
| DKP-6 | DOPC | Composition-2 | Process-4 | 75 | 0.080 | 91 |
| DKP-6 | DOPE | Composition-2 | Process-4 | 78 | 0.072 | 93 |
| DKP-6 | DEPE | Composition-2 | Process-4 | 84 | 0.099 | 91 |
| DKP-6 | DOPC | Composition-3 | Process-4 | 71 | 0.103 | 87 |
| DKP-6 | DOPE | Composition-3 | Process-4 | 76 | 0.143 | 94 |
| DKP-6 | DEPE | Composition-3 | Process-4 | 76 | 0.120 | 95 |
| DKP-14 | DOPE | Composition-4 | Process-1 | 108 | 0.161 | 71 |
| DKP-14 | DEPE | Composition-4 | Process-1 | 125 | 0.132 | 97 |
| DKP-14 | DOPE | Composition-4 | Process-2 | 117 | 0.150 | 85 |
| DKP-14 | DEPE | Composition-4 | Process-2 | 120 | 0.126 | 97 |
| DKP-14 | DOPE | Composition-4 | Process-3 | 114 | 0.147 | 92 |
| DKP-14 | DEPE | Composition-4 | Process-3 | 117 | 0.114 | 97 |
| GA-15 | DOPE | Composition-3 | Process-4 | 75 | 0.108 | 88 |
| GA-15 | DEPE | Composition-3 | Process-4 | 89 | 0.127 | 93 |
| GA-16 | DOPE | Composition-3 | Process-4 | 81 | 0.112 | 94 |
| GA-16 | DEPE | Composition-3 | Process-4 | 78 | 0.130 | 93 |
| MC-3 | DSPC | Composition-1 | Process-4 | 93 | 0.087 | 94 |
| MC-3 | DOPC | Composition-1 | Process-4 | 107 | 0.100 | 96 |
| MC-3 | DOPE | Composition-1 | Process-4 | 99 | 0.112 | 93 |
| MC-3 | DEPE | Composition-1 | Process-4 | 109 | 0.117 | 96 |
| MC-3 | DSPC | Composition-2 | Process-4 | 90 | 0.144 | 92 |
| MC-3 | DOPC | Composition-2 | Process-4 | 102 | 0.139 | 87 |
| MC-3 | DOPE | Composition-2 | Process-4 | 103 | 0.150 | 89 |
| MC-3 | DEPE | Composition-2 | Process-4 | 105 | 0.131 | 97 |

**Figure S6: Synthesis of** **DKF-6**

1. **Scheme for the synthesis of the core**

**Step 1: Synthesis of 4-(*tert*-Butyl) 1-methyl (2-(((benzyloxy)carbonyl)amino)-4-(*tert*-butoxy)-4-oxobutanoyl)aspartate (3)**

To a solution of 4-(*tert*-butyl) 1-methyl aspartate **1** (39.8 g, 0.17 moles) in 750 mL pyridine at 0 °C was slowly added a solution of 4-(*tert*-butyl) 1-(2,5-dioxopyrrolidin-1-yl) ((benzyloxy)carbonyl)aspartate **2** (70.0 g, 0.17 moles) in 250 mL DMF over a period of 3 h, and the reaction mixture was brought to room temperature and stirred overnight. After pyridine was removed under vacuum, the solution was poured into ice, and white solid was formed. The solid was filtered and triturated with 250 mL water. After filtration and drying, 4-(*tert*-butyl) 1-methyl (2-(((benzyloxy)carbonyl)amino)-4-(*tert*-butoxy)-4-oxobutanoyl)aspartate was obtained as white solid (80.0 g, 94 %).

**Step 2: Synthesis of 4-(*tert*-Butyl) 1-methyl (2-amino-4-(*tert*-butoxy)-4-oxobutanoyl)aspartate (4)**

To a solution of 4-(*tert*-butyl) 1-methyl (2-(((benzyloxy)carbonyl)amino)-4-(*tert*-butoxy)-4-oxobutanoyl)aspartate **3** (80.0 g, 0.16 moles) in 500 mL methanol and 150 mL dichloromethane was added palladium on carbon (10 wt %, 1.7 g), and the resulting mixture was stirred at room temperature under hydrogen atmosphere at 20 psi for 2 days. The reaction mixture was filtered through Celite, and the cake was washed with 25% methanol in dichloromethane. After concentration, 4-(*tert*-butyl) 1-methyl (2-amino-4-(*tert*-butoxy)-4-oxobutanoyl)aspartate was obtained as brown oil (30.0 g, 52 %).

**Step 3: Synthesis of Di-*tert*-butyl 2,2'-(3,6-dioxopiperazine-2,5-diyl)diacetate (5)**

To a solution of 4-(*tert*-butyl) 1-methyl (2-amino-4-(*tert*-butoxy)-4-oxobutanoyl)aspartate **4** (30.0 g, 80 mmol) in methanol (1500 mL) and dichloromethane (400 mL) at 0 °C was added ammonium hydroxide (40 mL) dropwise over a period of 1 h, and the reaction mixture was stirred at room temperature overnight. The suspension was concentrated to 500 mL and filtered. The solid was dried to get di-*tert*-butyl 2,2'-(3,6-dioxopiperazine-2,5-diyl)diacetate as white solid (21.5 g, 80 %).

**Step 4: Synthesis of 2,2'-(3,6-Dioxopiperazine-2,5-diyl)diacetic acid**

To a solution of di-*tert*-butyl 2,2'-(3,6-dioxopiperazine-2,5-diyl)diacetate **5** (28.5 g, 83 mmol) in 600 mL dichloromethane at 0 ˚C was added hydrochloric acid (4 M in dioxane, 200 mL) and the reaction mixture was stirred at room temperature for 2 days. The solid was filtered and triturated with dichloromethane to get 2,2'-(3,6-dioxopiperazine-2,5-diyl)diacetic acid as white solid (19.0 g, quantitative).

1. **Scheme for the synthesis of side chain 1,1'-((4-mercaptobutyl)azanediyl)bis(dodecan-2-ol)**

**Step 1: Synthesis of 2-(4-(Tritylthio)butyl)isoindoline-1,3-dione (7)**

To a mixture of sodium hydride (30 g, 1.08 mole, 60% dispersion in mineral oil) in 600 mL *N*, *N*-dimethylformamide, was added triphenylmethanethiol (200 g, 0.724 mole) in portions at 0 ^o^C. After stirring for 1 h, a solution of *N*-(4-bromobutyl)phthalimide **6** (204.3 g, 0.724 mole) in 400 mL *N*, *N*-dimethylformamide was added slowly, and the resulting mixture was allowed to warm slowly to room temperature and stirred overnight. The reaction mixture was poured into 6 L ice-cold water, and then the mixture was filtered and washed with water. The solid was dissolved in ethyl acetate and washed with brine. The organic layer was dried over Na_2_SO_4_ and concentrated to give the desired product as white solid (342 g, 98%), which was used for the next step without further purification.

**Step 2: Synthesis of 4-(Tritylthio)butan-1-amine (8)**

A mixture of 2-(4-(tritylthio)butyl)isoindoline-1,3-dione **7** (172 g, 0.36 mole) and hydrazine hydrate (75 mL, 1.80 mole) in ethanol (2.5 L) was heated under nitrogen atmosphere to gentle reflux overnight. After cooled to room temperature, the reaction mixture was filtered through Celite, and then washed with ethanol. The combined filtrate was concentrated under reduced pressure, and the residue was dissolved in chloroform. After stirring for 15 min, the mixture was filtered, and the filtrate was concentrated to afford the desired product as white wax (99.8 g, 80%), which was used for next step without purification.

**Step 3: Synthesis of 1,1'-((4-(Tritylthio)butyl)azanediyl)bis(dodecan-2-ol) (10)**

A mixture of 4-(tritylthio)butan-1-amine **8** (40 g, 0.115 mole) and 1,2-epoxydodecane **9** (63.4 g, 0.345 mole) in 500 mL isopropanol was heated under nitrogen atmosphere to gentle reflux for 20 h. The reaction mixture was concentrated, and the crude was purified by flash column chromatography (SiO_2_: 0-100% ethyl acetate in hexane gradient) to give the desired product as white solid (59 g, 71%).

**Step 4: Synthesis of 1,1'-((4-Mercaptobutyl)azanediyl)bis(dodecan-2-ol)**

To a solution of 1,1'-((4-(tritylthio)butyl)azanediyl)bis(dodecan-2-ol) **10** (59 g, 82 mmol) and triethylsilane (22.8 mL, 164 mmol) in 400 mL dichloromethane, was added trifluoracetic acid (67 mL, 82 mmol) dropwise at 0 °C, and then the resulting mixture was stirred at room temperature overnight. The reaction mixture was concentrated under reduced pressure, and the residue was dissolved in CH_2_Cl_2_. The solution was washed with 2 M aqueous sodium carbonate (4 x) until the aqueous layer pH >8, and then washed with brine. The organic phase was dried over Na_2_SO_4_. After filtration and concentration, the crude was purified by flash column chromatography (SiO_2_: 0-100% ethyl acetate in hexane gradient) to give the desired product as pale yellow oil (29.1 g, 74%).

1. **Scheme for the synthesis of DFK-6**

**Synthesis of *S,S*'-Bis(4-(bis(2-hydroxydodecyl)amino)butyl) 2,2'-(3,6-dioxopiperazine-2,5-diyl)diethanethioate (DFK-6)**

To a solution of 2,2'-(3,6-dioxopiperazine-2,5-diyl)diacetic acid **cDD** (0.96 g, 4.17 mmol) in 30 mL DMF was added a solution of 1,1'-((4-mercaptobutyl)azanediyl)bis(dodecan-2-ol) **TIM-4-E12** (4.8 g, 10.1 mmol) in 30 mL dichloromethane, and then followed by addition of DMAP (1.0 g, 8.2 mmol). The reaction mixture was cooled to 0 °C, EDCI (3.2 g, 16.7 mmol) was added, and the resulting mixture was stirred at room temperature overnight. The volatile was removed under vacuum, and the crude was purified by flash column chromatography (column: pretreated, neutralized silica gel 120 g, eluent: hexane and ethyl acetate 0-100%) to get *S,S*'-bis(4-(bis(2-hydroxydodecyl)amino)butyl) 2,2'-(3,6-dioxopiperazine-2,5-diyl)diethanethioate as white foamy solid (2.03 g, 42%). ^1^H NMR (300 MHz, CDCl_3_) δ 6.92 – 6.59 (m, 2H), 4.41 (d, 2H), 3.75 – 3.50 (m, 4H), 3.38 (d, 2H), 3.23 – 2.78 (m, 8H), 2.66 – 2.48 (m, 4H), 2.48 – 2.24 (m, 6H), 1.75 – 1.11 (m, 84H), 0.87 (t, 12H). APCI-MS analysis: Calculated C64H124N4O8S2 [M+H] = 1141.8, Observed = 1141.8.

**Figure S7: Synthesis of GA-16 (13)**

***Intermediate* 3:**

To a solution of triphenylmethanethiol (5.0 g, 18.08 mmol) in EtOH (40 mL) and water (40 mL) was added a solution (in 40 mL water) of NaOH (1.44 g, 36.16 mmol). The reaction mixture was stirred for 10 min and added a solution (in 40 ml EtOH) of 1,4-dibromobutane (3.65 g, 18.08 mmol) to reaction mixture. The reaction mixture was stirred for 4 hours at room temperature. The progress of reaction was monitored by TLC (5% EtOAc/hexanes). The reaction mixture was diluted DCM and aqueous sodium bicarbonate solution, the organic layer was washed with brine. The organic layer was dried over sodium sulphate, concentrated under vacuum to give crude compound. To the crude was added MeOH (15 mL) and stirred for 15 min at 0-10 ^o^C, the solid compound was filtered and dried under vacuum to give **3** (5.1 g, 69%) as a white solid. ^1^H NMR (400 MHz, CDCl_3_): δ 7.42-7.39 (m, 6H), 7.30-7.26 (m, 6H), 7.23-7.19 (m, 3H), 3.24 (t, 2H), 2.17 (t, 2H), 1.82-1.77 (m, 2H), 1.55-1.50 (m, 2H). LCMS: Purity 84.99 % (low ionization)

***Intermediate* 5:**

To a solution of **3** (5.0 g, 12.16 mmol) and **4** (3.16 g, 24.32 mmol) in ACN (75 mL) was added K_2_CO_3_ (6.72 g, 48.62 mmol). The reaction mixture was heated at 40 ^o^C for 48 hours. The reaction progress was monitored by TLC (2.5% MeOH in DCM)). The reaction mixture was cooled to room temperature and filtered. The filtrate was concentrated under vacuum to give crude product. The crude was purified by flash chromatography (0 to 2.5 % MeOH in DCM) to give **5** (2.6 g, 46%) as a white solid. ^1^H NMR (400 MHz, DMSO-d6): δ 7.41 (d, 6H), 7.28 (d, 6H), 7.20 (t, 3H), 3.59 (t, 2H), 2.73 (brs, 1H), 2.53-2.39 (m, 10H), 2.20-2.14 (m, 4H), 1.41 (brs, 4H). LCMS: Purity 98 %; *ESI-MS analysis:* Calculated C_29_H_37_N_2_OS, [M+H] = 461.26, Observed = 461.29

***Intermediate* 7:**

To a solution of **5** (0.5 g, 1.09 mmol) in DCM (8 mL) were added **6** (0.84 g, 1.19 mmol) in DCM (7 mL), EDC (0.31 g, 1.63 mmol), DMAP (27 mg, 0.22 mmol), DIPEA (0.38 mL, 2.17 mmol) and stirred at room temperature for 14 hours. After completion of the reaction as monitored by MS. The reaction mixture was diluted with DCM washed with NaHCO_3_ solution, water and brine. The organic layer was dried over anhydrous Na_2_SO_4_, concentrated, and the crude compound was purified (eluent: 20% EtOAc in hexanes) to obtain pure compound **7** as a color less oil (0.75 g, 60%). It was confirmed by MS analysis. *ESI-MS analysis:* Calculated C_69_H_120_N_3_O_4_SSi_2_, [M+H] = 1142.85, Observed = 1142.8

***Intermediate* 8:**

To a solution of **7** (0.75 g, 0.66 mmol) in DCM (6 mL) was slowly added TFA (6 mL) at room temperature and stirred at room temperature for 0.5 hour. To that triethylsilane (0.13 mL, 0.82 mmol) was added slowly and stirred for 1 hour. After completion of the reaction as monitored by MS. The reaction mixture was concentrated to obtain crude product **8** (>0.59 g, quantitative). It was confirmed by MS analysis. *ESI-MS analysis:* Calculated C_50_H_106_N_3_O_4_SSi_2_, [M+H] = 900.74, Observed = 900.7

***Intermediate* 10:**

To a solution of **8** (0.59 g, 0.65 mmol) in MeOH (10 mL) was added **9** (0.22 g, 0.98 mmol) at room temperature and stirred for 2 hours. After completion of the reaction as monitored by MS. The reaction mixture was concentrated, and the crude compound was purified (eluent: 8% MeOH in DCM) to obtain pure product **10** (0.45 g, 68%). It was confirmed by MS analysis. ESI-MS analysis: Calculated for C_55_H_109_N_4_O_4_S_2_Si_2_, [M+H] = 1009.74; Observed = 1009.7

***Intermediate* 12:**

To a solution of **10** (0.418 g, 0.41 mmol) and **11** (0.529 g, 0.83 mmol) in chloroform was added triethylamine (0.34 ml, 2.44 mmol) and allowed to react at room temperature for 2.5 hours. After completion of the reaction, the reaction mixture was concentrated and purified to obtain **12** as colorless oil (0.400 g, 63% yield). ESI-MS analysis: Calculated for C_90_H_182_N_4_O_6_S_2_Si_2_, [M+H] = 1537.76; Observed = 1537.8

**Synthesis of GA-16 (13):**

To a 20 ml polypropylene scintillation vial was added **12** (0.40 g, 0.26 mmol, 1.0 eq) along with 4 mL of dry tetrahydrofuran. The vial was cooled to 0-5 ^o^C and HF/pyridine (1.3 mL, 51.27 mmol) was added dropwise. After addition, the reaction vial was allowed to warm to room temperature and stirred for 18 hours. Afterwards, the reaction mixture was cooled back to 0 ^o^C and neutralized with solid sodium bicarbonate solid, diluted with ethyl acetate, washed with NaHCO_3_ solution, water and brine. The organic layer was dried over anhydrous Na_2_SO_4_ and concentrated. The crude product was purified to obtain compound **13** (0.20 g, 59%). It was confirmed by ^1^H NMR and MS analysis. ^1^H NMR (400 MHz, CDCl_3_) 5.43 – 5.28 (m, 4H), 4.20 (t, 2H), 3.65 (br, 4H), 2.86 – 2.24 (m, 32H), 2.07 – 1.89 (m, 8H), 1.88–1.52 (m, 10H), 1.52 – 1.11 (m, 80H), 0.87 (t, 12H). ESI-MS analysis: Calculated for C_78_H_154_N_4_O_6_S_2_, [M+H] = 1309.23; Observed = 1309.8
